# Molecular dynamics of JUNO-IZUMO1 complexation suggests biologically relevant mechanisms in fertilization

**DOI:** 10.1038/s41598-023-46835-0

**Published:** 2023-11-20

**Authors:** Paulina Pacak, Carleen Kluger, Viola Vogel

**Affiliations:** 1 Department of Health Sciences and Technology, ETH Zurich, Zurich, Switzerland; 2 Lehrstuhl für Angewandte Physik and Center for NanoScience, Ludwig-Maximilians-Universität, München, Munich, Germany; 3 Present Address: Evotec München GmbH, Neuried, Germany

**Keywords:** Biophysics, Computational biology and bioinformatics, Structural biology

## Abstract

JUNO-IZUMO1 binding is the first known physical link created between the sperm and egg membranes in fertilization, however, how this initiates sperm-egg fusion remains elusive. As advanced structural insights will help to combat the infertility crisis, or advance fertility control, we employed all-atom Molecular Dynamics (MD) to derive dynamic structural insights that are difficult to obtain experimentally. We found that the hydrated JUNO-IZUMO1 interface is composed of a large set of short-lived non-covalent interactions. The contact interface is destabilized by strategically located point mutations, as well as by Zn^2+^ ions, which shift IZUMO1 into the non-binding “boomerang” conformation. We hypothesize that the latter might explain how the transient zinc spark, as released after sperm entry into the oocyte, might contribute to block polyspermy. To address a second mystery, we performed another set of simulations, as it was previously suggested that JUNO in solution is unable to bind to folate despite it belonging to the folate receptor family. MD now suggests that JUNO complexation with IZUMO1 opens up the binding pocket thereby enabling folate insertion. Our MD simulations thus provide crucial new hypotheses how the dynamics of the JUNO-IZUMO1 complex upon solvation might regulate fertility.

## Introduction

The successful fertilization in mammals requires sperm capacitation, acrosome reaction, sperm penetration of the zona pellucida, specific sperm-egg recognition and finally membrane fusion with the egg^1,2^. The sequence of all those events must be highly specific, precisely coordinated in time and space, and be species-selective to ensure that only one sperm fertilizes the egg. Once a sperm has entered the perivitelline space of an egg, the first known specific physical link is formed between the two opposing membranes, more specifically between the two proteins, IZUMO1 and JUNO. IZUMO1 is a trans-membrane protein presented on the sperm surface^3^, while its counterpart, JUNO (also called IZUMO1 Receptor, IZUMO1R) is a GPI (glycosylphosphatidylinositol)-anchored membrane protein on the surface of the egg^4^. Their transient complexation is thought to initiate the recognition and adhesion process between the two gametes, possibly triggering their fusion. No clear mechanism has been identified so far, although different interaction models were proposed^5–8^. The possibility of a fusogenic activity of IZUMO1 has been discussed, but the evidence is still marginal. Experiments on normally non-fusing, innate HEK293 cells showed that simply the expression of JUNO and IZUMO1 is not enough to initiate the membrane fusion process^4^, despite recent reports that IZUMO1 independently of JUNO might serve as unilateral fusogen^9^. A few other additional molecular players were suggested to be crucially involved in this process^10–13^, yet their exact binding partners in the context of inter-gamete interactions have not been established^8^. Also, first efforts to identify successful JUNO-IZUMO1 interface inhibitors have been undertaken, regretfully, none of the small molecule candidates were confirmed to block this interaction^14^. This all suggests that far too little information of the hydrated structures is available to understand the crucial first steps of gamete fusion.

Given the lack of a conclusive molecular picture how the JUNO-IZUMO1 binding might initiate gamete fusion, we performed all-atom molecular dynamics (MD) and simulated the proteins alone, or in a complex with each other, in a box filled with explicit water molecules and ions. Such simulations allow to extract information how hydration of known protein crystal structures might change the dynamics of the JUNO-IZUMO1 interactions, thereby providing insights into the locations and lifetimes of critical bonds, information that is not easily accessible via traditional experimental methods of structural biology. MD is thus particularly well-suited to observe the time-dependent changes of the protein complexation and to capture new biologically relevant structural alterations^15,16^.

Our MD simulations were performed on the human JUNO-IZUMO1 complex, hydrated in explicit water, and for the proteins alone, using the wild type proteins whose structures have been recently resolved via crystallization^6,7^. To validate the predictive power of our MD simulations, we have also simulated the complex after introducing two in silico point mutations. The first one is known to abolish the JUNO-IZUMO1 interaction in vitro: IZUMO1’s TRP148ALA located in the binding interface^6,7^. The second is JUNO’s fertility-relevant mutation HIS177GLU^17^. As IZUMO1 in the crystal structures has been seen in two conformations, one that has a “straight” conformation like in the complex with JUNO^7^, and the second, where IZUMO1 adapts a bent conformation, also referred to as the “boomerang” conformation^6^. We asked how the shape of IZUMO1 might impact its binding to JUNO and whether the presence of divalent ions might stabilize one versus the other conformation. As the fusion of the first sperm with an egg is known to induce a “zinc spark”^18,19^, characterized by the rapid release of Zn^2+^ by the egg, our focus was then to study the interaction between Zn^2+^ ions and IZUMO1, and whether it could potentially play additional roles in fertilization. Looking into this question is also important considering the role of Zn^2+^ ions in regulating a sperm’s response to progesterone via binding to a yet unknown sperm receptor^20^.

Finally, we addressed another curiosity too. JUNO, a member of the folate receptor (FR) family, also referred to as Folate Receptor 4, did surprisingly not bind to folate in solution when tested alone in vitro^4,21^, despite the known importance of folate for female fertility^22–24^. We thus asked using well established computational protocols, whether folate binding to JUNO might involve mechanisms similar to those found in Folate Receptor *α* (FR*α*; PDB 4LRH)^25–27^ but triggered by JUNO-IZUMO1 complexation, and were indeed able to witness events where folate was slipping into JUNO’s binding pocket.

Together, these in silico experiments enabled us for the first time to take into account dynamic changes of the JUNO-IZUMO1 complex, as imposed by hydration in explicit water. Our MD data allowed us to derive new structural hypotheses how the specific gamete binding is regulated, and thus contribute in unique ways to unravel the mystery of fertilization.

## Results

For the atomistic modeling, the modeled crystal structures^6,7^ of JUNO or IZUMO1, either alone, or in complex with one another, were solvated in explicit TIP3 water, neutralized and placed in a physiological salt concentration for equilibrium MD simulations at 310 K (see Methods for more details). The solvent molecules are not shown in the Figures and Videos. To account for the stochastic nature of MD simulations, we repeated each simulation 10 times (unless indicated differently, for details see Table S1). The results were analyzed by various python-based scripts to assess the conformational stability of the proteins (for detailed information see Supplementary Information Figures S7–S16 ) and via PyContact^28^ to understand the non-covalent interactions.

### The hydrated JUNO-IZUMO1 complex got stabilized by a large network of short-lived interactions

**Figure 1:**
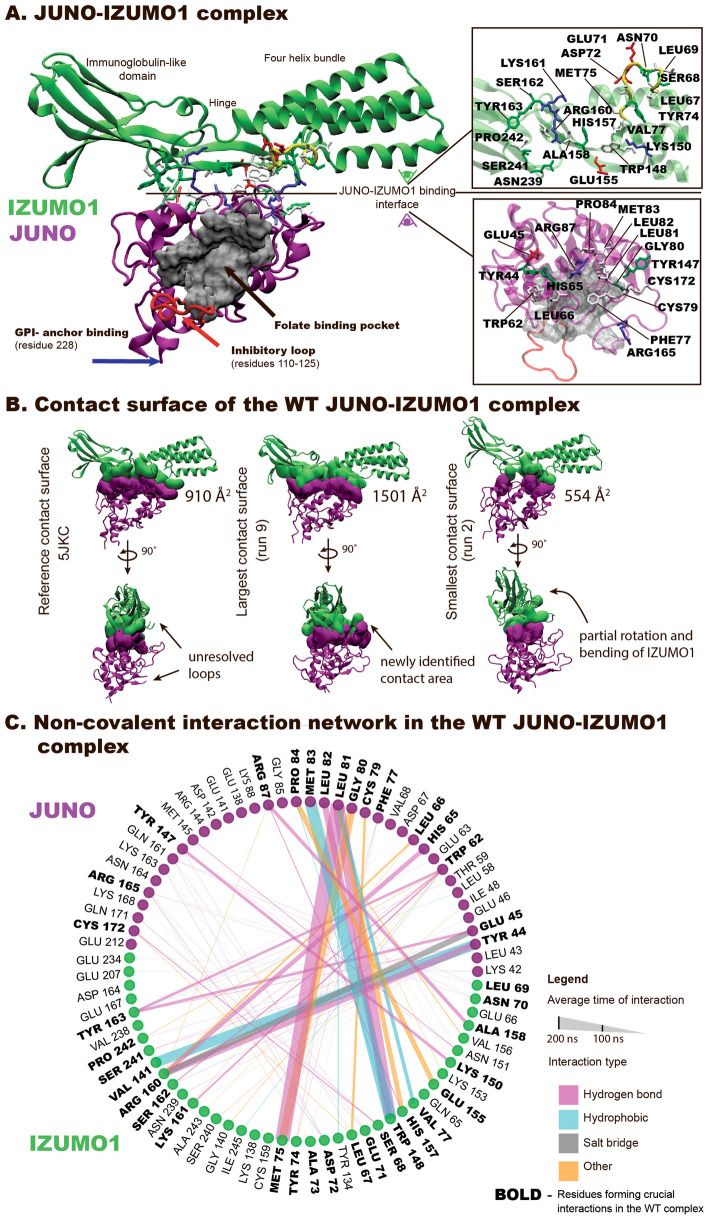
Interaction interface of the human WT JUNO-IZUMO1 complex as it fluctuates in explicit water solution. (**A**) Model of the JUNO-IZUMO1 complex used in this study (water molecules and ions are omitted in the visualization). For JUNO, only residues 20–228 are considered (excluding the signal peptide). For IZUMO1, only its extracellular part comprising residues 22–254 is used. The largest unresolved parts in the crystal structure of the complex included the inhibitory loop of JUNO (residues 109–124, marked in red), which has a rather intriguing position, right on the side of JUNO’s putative central folate binding pocket, as well as the loop between residues 68 and 75 of IZUMO1 (marked in yellow). Residues of the binding interface are shown in the stick representation and are colored based on their type (green: polar, red: acidic, blue: basic, white: non-polar). Regions of special interest are marked with arrows. (**B**) Representative contact interface between JUNO and IZUMO1. The area as seen in the crystal structure is shown on the left. It contains unresolved/missing regions. The frame with the maximum contact area achieved in equilibrium simulations (middle) reveals previously unidentified contributing residues between the two proteins. The binding interface is mostly defined by transiently forming hydrogen bonds. The frame with minimal contact area (right) shows partial bending and rotation of the IZUMO1. (**C**) Inter-protein interactions observed across all simulations of the WT JUNO-IZUMO1 complex (averaged over 10 runs). Residues highlighted in (**A**) are shown in bold. Only few non-covalent interactions remained intact throughout the simulation time of 200 ns. Most are short lived and can rapidly change binding partners. Interactions extracted using PyContact^28^ with default settings. The threshold for hydrogen bonds was 2.5 Å and an interaction mean score bigger than 1. See also Movies S1 and S2 for the dynamics of the JUNO-IZUMO1 interface.

When analyzing how solvation of the complex impacts the total number of interactions formed between JUNO and IZUMO1, an overall increase in inter-protein contacts was seen with respect to the known crystal structure (Fig. 1). During the 200 ns simulations, adjustment of the relative JUNO and IZUMO1 positions typically occurred, driven mainly by the formation of new interactions on the interface between IZUMO1’s loop at residues 67–75 (between helices *α*2 and *α*3), that were in part missing in the crystal structure, and JUNO’s loop at residues 161–168 as illustrated in Movies S1 and S2. The contact area between the proteins rapidly fluctuated both within a single run and between the runs with standard deviations per run in the range of 60–100 Å^2^ (Figs. 1B, S20 and Table S2). The smallest and largest contact areas observed were 554 Å^2^ and 1501 Å^2^, respectively, as compared to 842 Å^2^^7^ or 910 Å^2^^6^ for the two crystal structures. MD furthermore revealed that the network of interactions at the binding interface consists of a large cluster of mostly short-lived (ns) contacts (Fig. 1C), containing up to 35 non-covalent interactions on average per simulation, but the number of simultaneously formed contacts varied between different runs (Fig. S21). In total, there are 28 residues on JUNO involved in the interaction with IZUMO1 in at least one run, spanning through helices *α*1, *α*2, part of helix *α*3 and neighboring loops. On IZUMO1, there are 33 different residues, placed mostly in its central hinge region and on both sides of it: the loop between strands 8 and 9 of the Ig-like domain, and the loop between helices *α*2 and *α*3 on the side of helix bundle. This was in agreement with previous experimental studies that asked how point mutations of the JUNO-IZUMO1 interface tune the affinity, thereby revealing that residues GLU45 and LEU81 on JUNO and TRP148, HIS157 and ARG160 on IZUMO1 are essential for the binding to occur^6,7^. Other residues interacting in our simulations are marked in Fig. 1C.

Of the identified non-covalent interactions, 17 have been formed at least temporarily in all runs, including eight hydrogen bonds (Fig. S22). Only 3–7 interactions are persistent throughout the entire time window in each run. Mean lifetimes (Fig. S21) and exemplary runs with the highest and lowest number of contacts, i.e. with 45 contacts (run #9) and 32 contacts (run #7), respectively, are shown in the Supplementary Figs. S22 and S23. Quantification of the bond network fluctuations in various runs of the JUNO-IZUMO1 complexes revealed that most of the interfacial contacts were highly dynamic, with individual bonds forming and breaking multiple times within the 200 ns time window. The quantifications were done by exploiting various in-house scripts (see “Methods”).

### IZUMO1 in explicit water freely fluctuated between two conformational states: straight and boomerang

**Figure 2:**
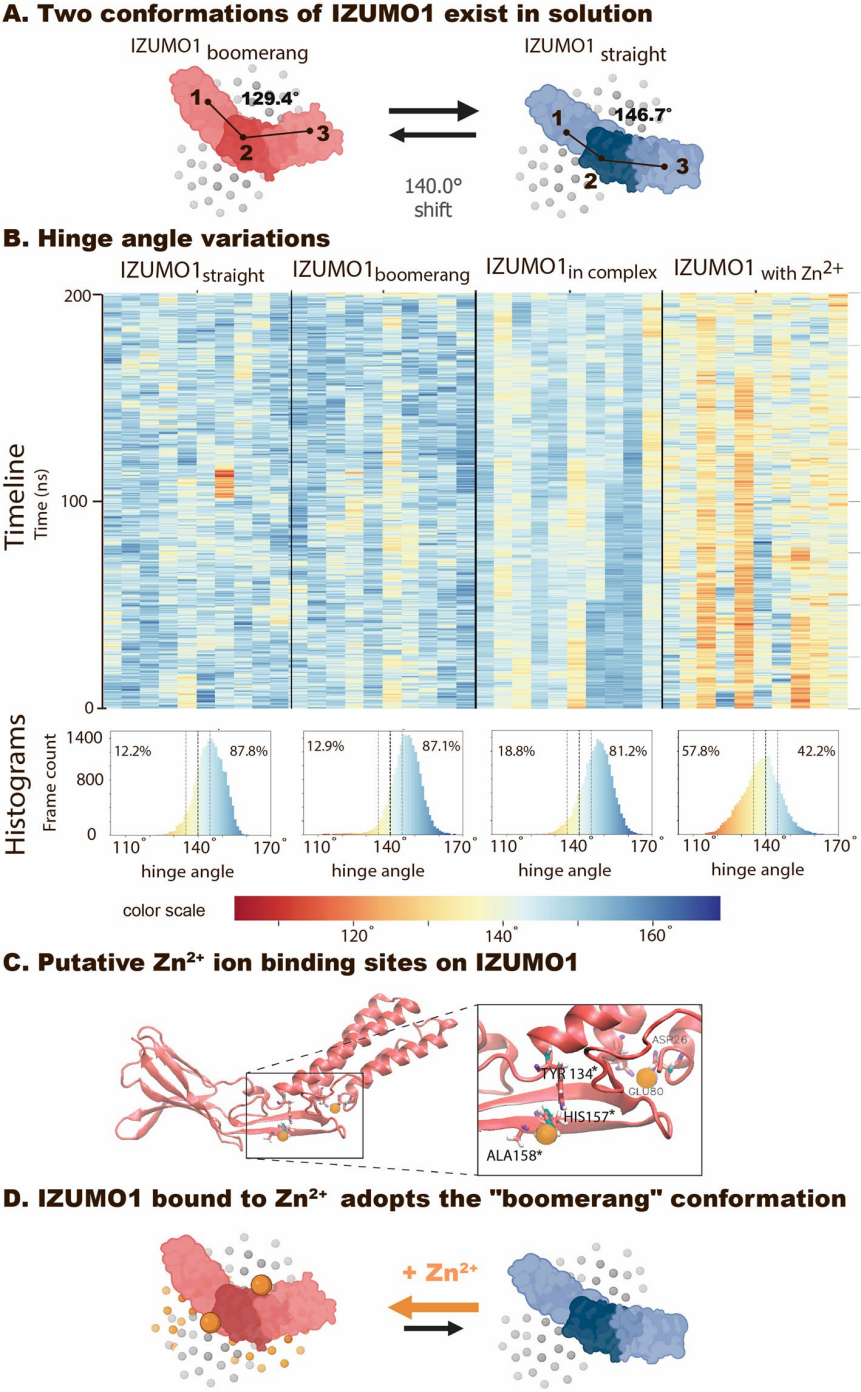
IZUMO1’s hinge angle fluctuations revealed by MD. (**A**) Schematic representation for two IZUMO1 conformations identified by crystallography: “boomerang” (red)^6^ and “straight” (blue)^7^. The fluctuating hinge angle between the center of mass (COM) of different IZUMO1 domains (COM_1_)–(COM_2_)–(COM_3_) was quantified as a function of time for all the replicas (shown in **B**). An empirical threshold was thereby set at 140° to distinguish between the “boomerang” and the “straight” conformations. (**B**) Timelines of hinge angle variations of individual trajectories are shown (200 ns) for four different systems, each with 10 replicas. Each frame is colored according to the value of the hinge angle. Below, the histograms of the hinge angles are shown for each condition (each bin corresponds to 1°). Percentage of frames where IZUMO1 is present in the “boomerang” (angle < 140°) or “straight” (angle > 140°) is also marked. (**C**) Snapshot of representative IZUMO1-Zn^2+^ binding sites. Both ions remained bound in runs #2, #3, #5, #7, #8, #9, which is marked in B by accumulation of red frames for respective columns. See also Movie S9. (**D**) Schematic representation of the conformational shift of IZUMO1 towards the “boomerang” conformation as induced by the binding of Zn^2+^ ions (made with BioRender.com).

The structure of IZUMO1 alone has been captured by crystallography in two different conformations, named here “straight”^7^ and “boomerang”^6^. They differ in the relative positions of the Ig-like and 4-helix bundle domains that are connected by a hinge region (shift of around 20 Å as reported in^6^). When in complex with JUNO, IZUMO1 has only been crystallized in the “straight” conformation. To test whether IZUMO1 can fluctuate between these two conformations in solution, we conducted 200 ns simulations of IZUMO1 alone in both conformations, as well as simulations of the JUNO-IZUMO1 complex, each repeated 10 times. Indeed, IZUMO1 fluctuated between the “straight” and “boomerang” conformations multiple times, independently of the starting conditions (Movie S9). The movement was mostly localized in the hinge region, causing pronounced bending motion, while no other significant conformational shifts occurred in the adjacent domains (except for movement in one loop at residues 67-71 in certain simulations, for a more detailed conformational analysis, see Supplementary Information and Figs. S7, S9, S13). To evaluate IZUMO1’s fluctuations, the hinge angle defined as the angle between the centers of mass (COM) of the three different domains of IZUMO1 (4HB, hinge and Ig-like domain) was used, as well as a threshold angle of 140° to mark the transition point between the two conformations. In the crystal structures, the “straight” (PDB 5F4T) and “boomerang” (PDB 5JK9) conformations displayed the hinge angle of 146.7° and 129.4°, respectively (Fig. 2A). The time evolution of the hinge angle for IZUMO1 alone confirms that fluctuations occur in both directions, whereby the “boomerang” conformation can be reached spontaneously when starting from the “straight” conformation, and vice versa, with similar rates (Fig. 2B). For all 10 trajectories of 200 ns each, we have observed 1766 and 1591 shifts for IZUMO1 starting in either the “boomerang” or the “straight” conformation, respectively (Fig. 2, Movie S9). The overall preference towards the “straight” conformation should be noted though, as only 12.2% and 12.9% of the time is spend in the “boomerang” conformation. Once the IZUMO1 was in complex with JUNO (IZUMO1_*in complex*_), less extreme hinge angles were observed overall (Fig. 2B, histograms), but at the same time more frames could be classified as “boomerang” (18.8% of time) according to the arbitrary threshold of 140°. We verified the results with unbiased clustering of all IZUMO1 structures (Fig. S17) and confirmed that only around 12% of the time is spent in the true “boomerang” conformation, while a third state, “intermediate”, was assigned to the frames previously characterized as “boomerang” or “straight” (see Supplementary Information S1 for details).

The largest contact area between JUNO and IZUMO1 was seen in run #9, which also showed the highest number of stable interactions (Fig. S22) and was almost continuously in the "straight" conformation (Fig. 2). This poses the question whether the “boomerang” conformation of IZUMO1 is unfavorable for JUNO binding. For a discussion regarding the differences between our MD predicted distribution of IZUMO1’s structure in solution and some experimental findings, please see the Methods.

Please note that our theoretical predictions somewhat contradict the previously suggested hypothesis that IZUMO1 in solution exists preferentially in the “boomerang” conformation, as concluded from small-angle X-ray scattering and deuterium exchange mass spectrometry^6,7^. Differences in experimental methods and conditions such as single molecule changes as observed here versus population wide data analyses^29,30^ and varying physio-chemical factors (pH, temperature of 283 K in experiments vs 310 K in simulations) and insensitivity to the fast time scales that we are simulating here, may account for these discrepancies.

### IZUMO1’s boomerang conformation did not stabilize when docked to JUNO

**Figure 3:**
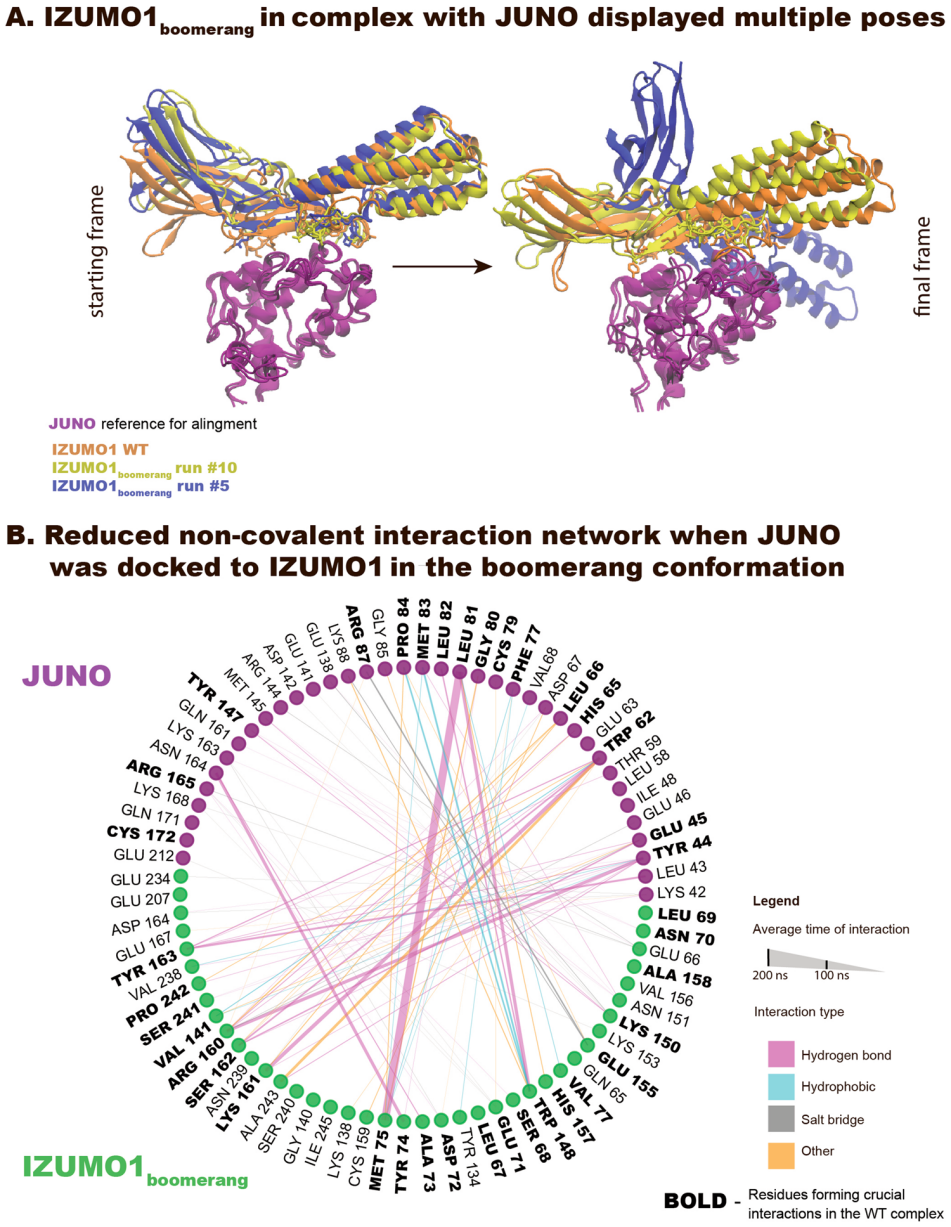
Alterations in the interaction network of JUNO with IZUMO1 in its “straight” versus “boomerang” conformation. (**A**) Snapshots of the complex aligned on the JUNO structure (purple) of the initial (left) and final (right) frames for the exemplary runs for the IZUMO1 WT complex (orange) and for IZUMO1’s “boomerang” runs: yellow, where most of the interactions were preserved (run #10, see also Movie S6) and blue, where the majority of the interactions were lost, causing a re-orientation of the relative positions of the proteins within the complex (run #5, see also Movie S7). Water molecules and ions are omitted for visualization. While the yellow and blue structures were closer in the overall alignment in the initial frames (both are “boomerang” structures), the lack of strong interactions around the Ig-like domain resulted in significantly different final positions. Different patterns emerged between the runs, involving new residues (see also Supplementary Information, Fig. S21). (**B**) Inter-protein interaction network. The interface observed for the WT complex (bold residues) is partially disrupted, while the formation of new and transient interactions can be observed.

By docking IZUMO1’s “boomerang” conformation to JUNO’s known binding interface and running 10 simulations of 200 ns each, we asked whether IZUMO1’s straightening is stimulated by JUNO and can be observed computationally too. Figure 3A shows the various outcomes, from an almost complete JUNO-IZUMO1 interface recreation (Fig. S27 and Movie S6) to partial unbinding and reorientation of IZUMO1 with respect to JUNO’s binding interface (Fig. S28 and Movie S7). Most of the interactions that were present for the JUNO-IZUMO1_*straight*_ complex, are lost after the equilibration phase of the JUNO-IZUMO1_*boomerang*_ complex (Figs. 3B, S21). While the interaction interface on the side of the Ig-like domain is usually preserved by at least 5 residues, the “boomerang” conformation of IZUMO1 strongly disrupts the region neighboring with the Ig-like domain of IZUMO1 (Fig. 3A). This resulted in the weak pivoting points (H-bonds: TYR44_*JUNO*_-ARG160_*IZUMO*1_, GLU45_*JUNO*_-ARG160_*IZUMO*1_), that are enough to keep the binding partners in proximity within the simulated time window, but do not allow for the recreation of the optimized, large binding interface within the simulated time window (Fig. S28 and Movie S7). Formation of an additional H-bond, which was observed on the Ig-like side of the interface (TRP62_*JUNO*_- SER162_*IZUMO*1_) in only a few simulations, is sufficient to temporarily recreate stable binding (Fig. S28 and Movie S6). However, even when the essential interactions are partially reformed, their average lifetime drops below 20% of the simulation time (400/2000 frames), and some of them are only transiently present (Fig. S27). This suggests that the “boomerang” conformation is incompatible with JUNO-IZUMO1 complexation.

### Zinc^2+^ ions shifted IZUMO1 preferentially toward its boomerang conformation

As heavy ions are known to interfere with fertility^31,32^, we asked whether the presence of heavy ions might stabilize IZUMO1 in the “boomerang” conformation, thereby disrupting the formation of a large interface with JUNO by stabilizing IZUMO1 in the “boomerang” conformation. Using a Metal Ion-Binding Site Prediction and Docking Server^33,34^, we have identified potential ion binding sites on IZUMO1 for a few divalent ions, including Cu^2+^, Cd^2+^ and Zn^2+^ (Fig. S30). The same ions were docked to JUNO for comparison, but not investigated further (Fig. S31). For IZUMO1, two positions on each side of the hinge region were then chosen as potentially being relevant for steering the bending motion, as those two sites were common for different ions in the predicted structures (Fig. S30). The first is positioned close to the Ig-like domain of IZUMO1 and the other one at the base of the helical bundle, but still in the proximity to the hinge region. Zn^2+^ ions were then chosen because they can be, first, parameterized in the CHARMM force field^35^, second, are representative in size for other heavy ions (Table S3), and third, are known to play a role in fertility^31^. Running the simulations in the presence of Zn^2+^ ions is physiologically particularly important, as extracellular zinc is transiently abundant right after successful fertilization in the so-called transient “zinc spark” event^18,19^. To evaluate how the bending motion of IZUMO1 is impacted by the presence of multivalent ions, we repeated the same analysis of the hinge angle as for the previous conditions (Fig. 2B). When inserting two Zn^2+^ ions into those above-predicted locations in the “boomerang” conformation, IZUMO1 stayed preferentially in the “boomerang” conformation, whereby both Zn^2+^ ions remained bound in their predicted sites in 6/10 simulations (runs #2, #3, #5, #7, #8, #9). The IZUMO1-ion interactions are non-covalent. Each divalent ion binding site consists of 1 to 3 protein residues (Fig. 2C), while 5 or 6 water molecules coordinate with each Zn^2+^, which is not uncommon for hydrated Zn^2+^ ions^36^. In other replicas (4/10), only one of the ions placed near the hinge region unbound and subsequently sampled additional areas on the surface of the protein or diffused away into solution. We thus hypothesize that heavy ions can stabilize IZUMO1 in the “boomerang” conformation, thus disfavoring binding to JUNO.

### IZUMO1’s TRP148ALA mutant disrupted the binding interface, while JUNO’s HIS177GLU infertility mutant showed no allosteric effect on the JUNO-IZUMO1 binding interface

**Figure 4:**
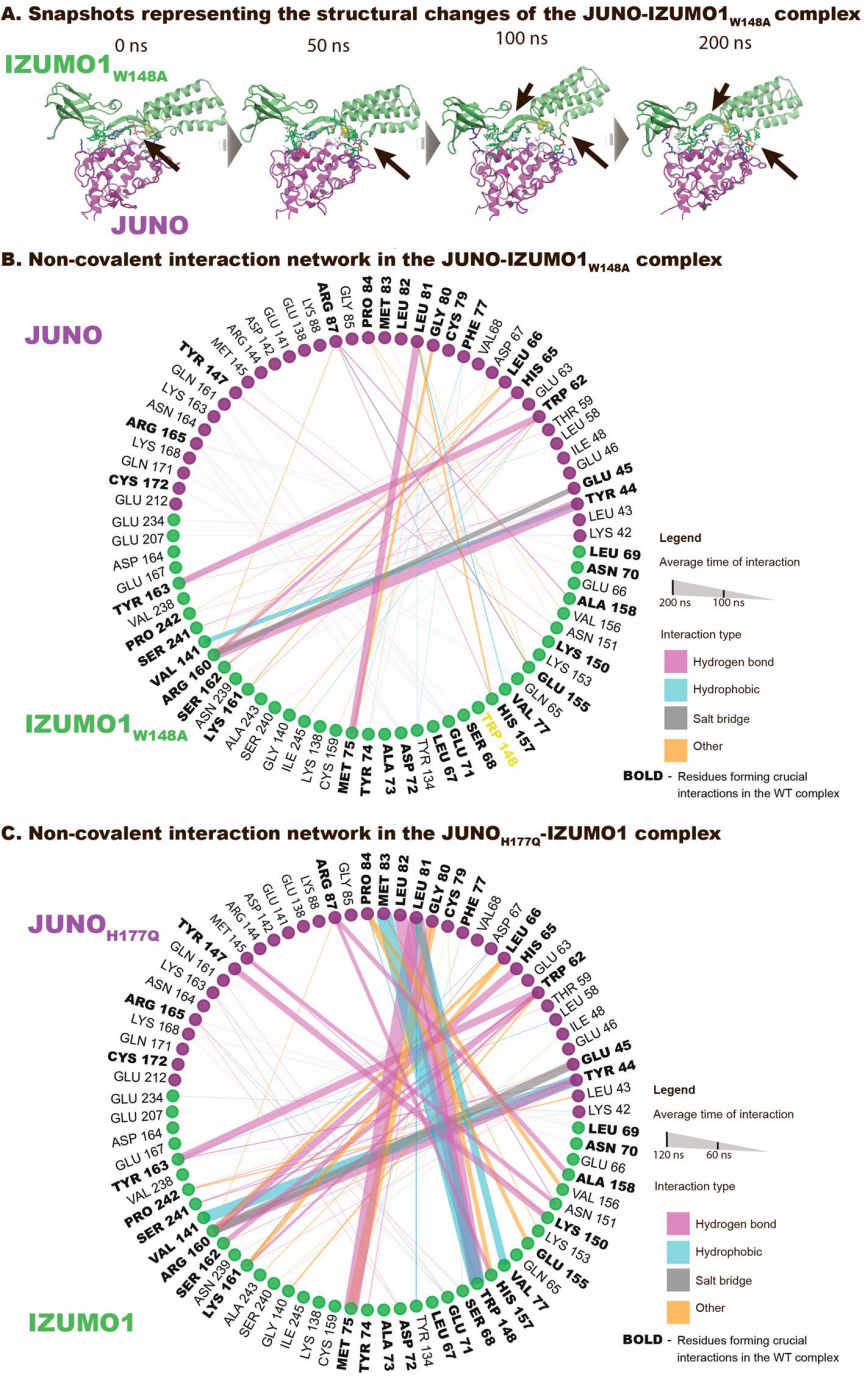
Dynamics of the non-covalent interaction network in the human JUNO-IZUMO1 complex with selected in silico mutations. (**A**) Exemplary snapshots of the mutated JUNO-IZUMO1 W148A complex at different time points (water molecules and ions are omitted for visualization). Residues involved in major interactions are marked. The breakage of crucial non-covalent interactions and the variations in the position of IZUMO1’s 68–71 loop of can be observed, leading to its partial unbinding. (**B**) Inter-protein interaction network for JUNO in complex with IZUMO1 W148A. All the interactions were shorter-lived than in the WT complex (see also Movie S4). (**C**) Inter-protein interaction network for JUNO H177Q in complex with IZUMO1 (total simulation time per run was 120 ns). Simulations revealed no differences when compared to the WT, suggesting that this mutation outside of the binding interface does not influence JUNO-IZUMO1 binding under equilibrium conditions (see also Movie S5). Interactions extracted using PyContact^28^ with default settings, whereby the threshold for hydrogen bonds was 2.5 Å and an interaction mean score was bigger than 1.

IZUMO1’s TRP148 is one of the best-preserved residues of IZUMO1 among mammals and its mutation to ALA has been shown to abolish JUNO-IZUMO1 binding^6,7^. In an in silico control, we thus introduced this point mutation TRP148ALA to check whether the same effect could be observed in our simulations. This resulted in gradual unbinding of IZUMO1’s hinge region from JUNO (Figs. 4A, S25 and Movie S4) and overall deformations of IZUMO1’s structure. The mutation of TRP148 to ALA on IZUMO1 caused the disruption of the interaction network, particularly by lacking the crucial hydrogen bonds with JUNO’s residues LEU81 and LEU82, that are among the longest lived interactions found here for the WT complex (Figs. 4B, S21). Our simulation time was too short to achieve full unbinding, but the observed changes already point to the unfavorable effect of this mutation on JUNO-IZUMO1 binding and provided sufficient validation of the sensitivity of our model. We thus proceeded with another mutational in silico test. Since JUNO’s mutation of HIS177GLU is also associated with in vivo fertility failure^17^, yet this residue is not part of the known JUNO-IZUMO1 interface, we investigated whether it might be disruptive, potentially via allosteric effects on IZUMO1 binding. The in silico HIS177GLU mutation of JUNO, however, revealed no indications that the binding interface is impacted via a long-range allosteric mechanism (Figs. 4C, S21, S26 and Movie S5), at least within the observed time window.

### MD captured spontaneous binding of folate into JUNO’s binding pocket, but only when in complex with IZUMO1

**Figure 5:**
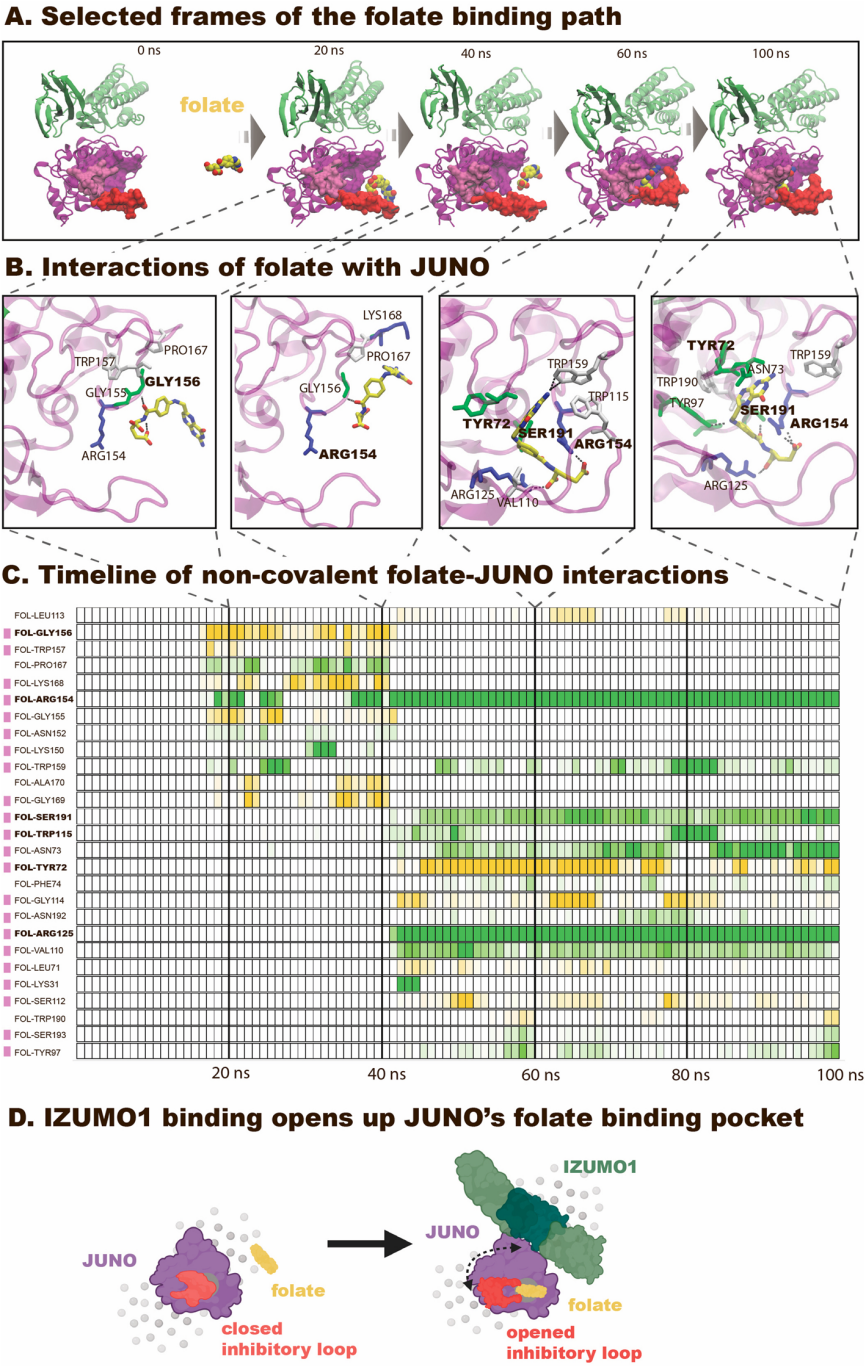
Spontaneous folate binding to JUNO observed in silico is enabled by complexation of JUNO and IZUMO1. (**A**) Representative snapshots of the successful folate binding trajectory in the 100 ns equilibrium MD simulation (run #1, Movie S12). Proteins are shown in cartoon representation (purple: JUNO, green: IZUMO1), residues around the central binding pocket of JUNO are shown as surface representation (red: residues 110–125, pink: 29–35, purple: pocket residues). Water molecules and ions are omitted for visualization. In the first frame, folate is placed 50 Å away from the complex. After shortly exploring the solvent around the binding complex, folate subsequently approached the inhibitory loop, creating a first interaction with GLY156. Subsequently, it gradually formed new bonds with ARG154 and was sliding inside the binding pocket. (**B**) Magnified view of the main Folate-JUNO interactions. Protein residues are colored based on their type (green: polar, red: acidic, blue: basic, white: non-polar). Once bound to JUNO, folate stayed inside the binding pocket, but did not reach the full insertion depth. (**C**) Timeline of non-covalent interactions between folate (FOLT) and JUNO. Each box represents an equal time segment. Pink labels mark hydrogen bonds. The interaction scores are coded by color intensity and color-coded based on the nature of the interaction (green: side chain interaction; yellow-backbone interaction (made with PyContact^2^). (**D**) Schematic representation (made with BioRender.com). JUNO alone displayed the inhibitory loop in a closed conformation, thereby blocking access to the putative folate binding pocket. As a sperm binds to an egg in the process of fertilization, IZUMO1 binds to JUNO to form a complex that is transiently stabilized by a large cluster of fluctuating non-covalent interactions. In the presence of IZUMO1, JUNO’s inhibitory loop opened up, thereby enabling access to its central folate binding pocket, and at the same time increasing pocket’s volume. This allows to insert folate computationally, or alternatively perhaps the binding of other small molecules. See also Movies S10–S13 and Supplementary Information S1 for details.

Another puzzling aspect of JUNO’s structure, that we tackled by MD, is its proclaimed loss of folate binding ability^4,21^. We asked here computationally whether binding of folate to JUNO could be achieved if in complex with IZUMO1, which remains to be an untested experimental condition. A first hint came from our MD observation that JUNO’s TRP190 adjacent to the binding pocket adapts different orientations upon hydration compared to the crystal structure. Rather than permanently hindering access to the binding pocket^37^, we observed it to fluctuate (Fig. S1). To test our hypothesis, folate was placed 50 Å away from the JUNO-IZUMO1 complex in a water box, and the ligand was allowed to diffuse freely for 100 ns in 10 independent replicas. Indeed, we could capture one successful binding event where folate spontaneously inserted into the folate binding pocket, as well as a few partial, mostly unspecific binding events (for details see Supplementary Information S1). The stepwise insertion of folate into the binding pocket could be observed within 45 ns. Once bound, the ligand position was stable for the remaining simulation time, with the inhibitory loop partially blocking the free exit route (Fig. 5 and Movie S12). The folate in the observed pose was only partially embedded inside of JUNO (Fig. S2), not reaching as deep inside the pocket as it was reported for the FRα^38^. Folate is seen to be bent (Fig. 5 and Movie S12), supporting previous MD findings on FRα ligands showing that folate and its analogues are flexible molecules and can adapt various shapes upon binding^39^. In the remaining simulation time (to the total of 100 ns), no pose correction towards the known crystal structure was observed (Fig. S3). Interestingly, the successfully bound pose was similar to the one reported in the computational study of FRα^27^. None of the residues interacting with folate were previously observed as being directly involved in the JUNO-IZUMO1 interaction, and folate insertion in the binding pocket did not cause any noticeable changes in the JUNO-IZUMO1 interface during the simulation time (Figs. S6, S29 and Movie S8).

To further verify whether deeper insertion of folate into the binding pocket is possible and was dependent on IZUMO1’s presence, we have created two more sets of simulations where the intermediate state of the previously successful run (t  =  43 ns) was used as a starting point. In the first set, the JUNO-IZUMO1 complex is used and simulated in 10 repeats (Fig. S2) that we refer to as “refined”. In the second set with 5 repeats, the same relative orientation of JUNO and folate is used, but IZUMO1 is removed and JUNO alone is used instead (Fig. S4), whereby slight adjustments of the pose is made to account for sterical clashes (for details see Supplementary Information S1). In the “refined” binding set, two more binding events were observed: shallow insertion, similar to the one observed in the spontaneous binding (Fig. S3, run #8) and deep insertion, where folate reached the same depth as in the known FRα structure^38^ (Fig. S2 and Movie S11). In all observed pocket-binding events, JUNO’s charged residues ARG154 and GLY156 were involved in forming the very first JUNO-folate contact, thereby guiding folate’s way towards the center of the binding pocket. During the insertion, a maximum of eight hydrogen bonds were formed, as well as two salt bridges, and two π-stacking interactions (Fig. 5). Previous MD studies on FRα showed multiple intermediate steps of folate-FRα binding and concluded that a combination of hydrogen bonding, π-stacking, and van der Waals and Coulomb attraction need to be present simultaneously in order to accomplish specific binding^27^. Our best observed pose created a sparse network of interactions as shown in Fig. S2, even though some important residues were missing, when compared to the crystal structure of FRα^38^. In the simulations of folate with JUNO alone, the folate binding inside the pocket was not observed, however, in 4 out of 5 runs folate unsuccessfully screened the outer side of it (Figs. S3, S4 and Movie S13). Taken together, those in silico results suggest that JUNO’s putative folate binding pocket is rendered more accessible in the presence of IZUMO1. We hypothesize that the presence of IZUMO1 stabilizes the inhibitory loop at the entrance of JUNO’s folate binding pocket in an open configuration. Indeed, our comparative structural analyses of simulations of JUNO alone and in the complex with IZUMO1 show synergies between IZUMO1 binding and the accessibility of the central binding pocket (for details see Supplementary Information and Figs. S5, S6, S8, S10, S11, S12, S14, S15, S16, S18, S19 and Movies S10, S3).

## Discussion

**Figure 6:**
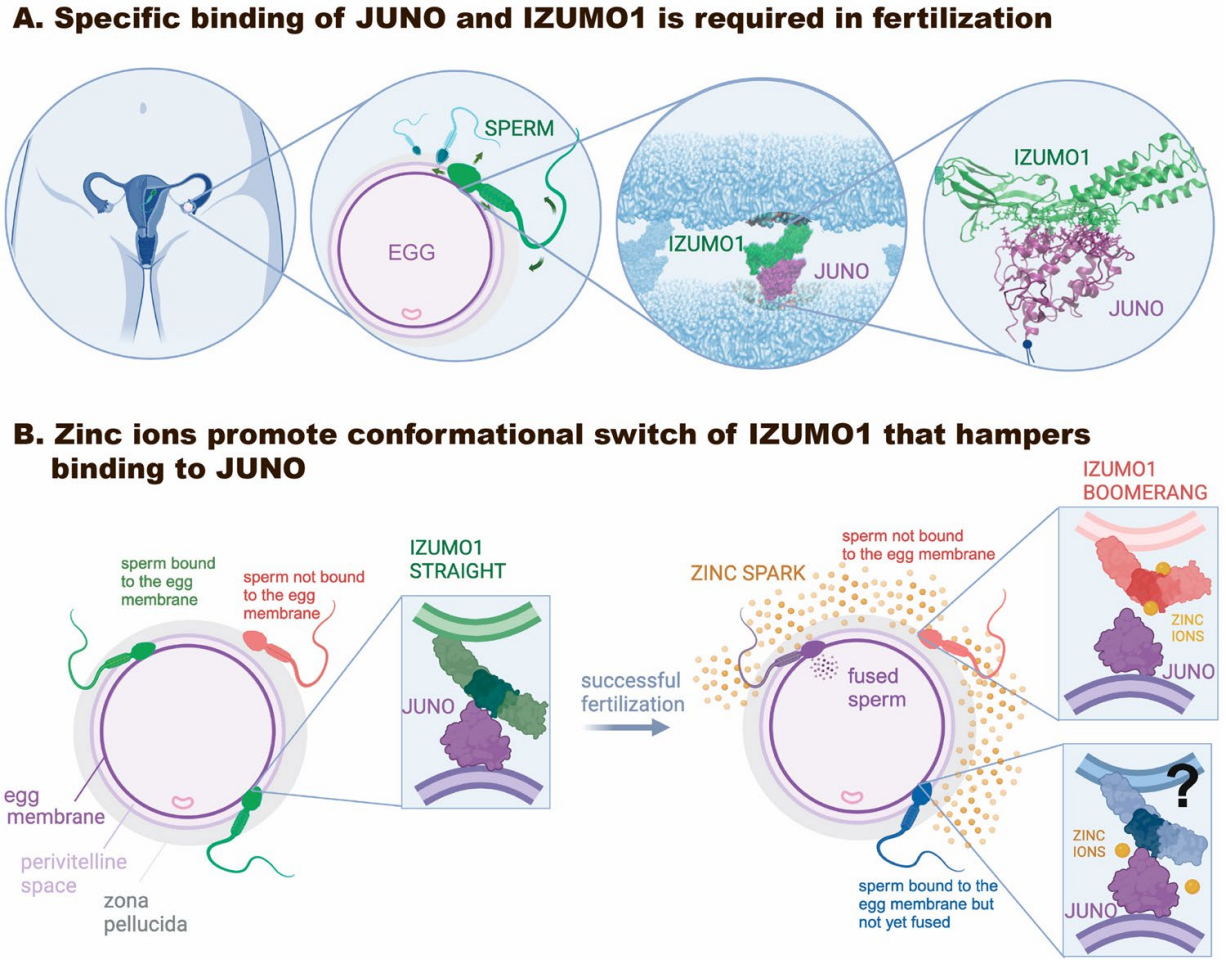
Potential biological implications. (**A**) Specific binding of JUNO to IZUMO1 is required for fertilization to proceed. Sperm introduced into the female reproductive track moves first through the uterus to the fallopian tubes, propelled by the vigorous movement of the sperm's flagellum in order to reach a released egg. Once the target is reached, it has to first cross the zona pellucida, undergo the acrosome reaction and finally approach the egg membrane and adhere to IZUMO1 creating the specific physical link. The JUNO-IZUMO1 complex has been crystalized^6,7^. (**B**) While multiple sperms typically reach an egg, only one can fertilize. For the JUNO-IZUMO1 complex to form, we show that IZUMO1 must remain in a “straight” conformation. After the first sperm fuses with the egg, zinc ions are released into the extracellular environment which gives rise to the so-called “zinc spark”^18,19^. Subsequent sperms that have not yet adhered to the membrane, get subsequently exposed to zinc ions, which we propose triggers a rapid transition of IZUMO1 into the “non-binding” boomerang conformation, thus disfavoring complexation with JUNO. This is hypothesized here to act in concert with other mechanisms that prevent polyspermy, thereby reducing the risk that other sperms can fuse with the already fertilized egg, before more robust polyspermy blocking mechanisms can get activated. Whether zinc ions can cause the unbinding of already formed JUNO-IZUMO1 complexes (e.g. on other membrane-bound sperms) is not known but suggested by our preliminary simulation (figure created with BioRender.com).

Our MD simulations using the high-resolution crystal structures^6,7^ hydrated in explicit water molecules, revealed detailed insights into the dynamics of the JUNO-IZUMO1 complex. As the physiologically relevant fertilization events evidently take place in water, our first crucial observation is that the JUNO- IZUMO1 interface got stabilized by a large network of short-lived contacts. Importantly, new residues that were not part of the binding interface in the crystal structure could interact with each other in the hydrated complex. This is significant, as the identification of additional residues can help to better target this interface. Center piece is a large network of non-covalent and short-lived bonds that stabilizes the interaction between JUNO and IZUMO1 (Fig. 1), and our simulations suggest functional design features of the complex that remained so far elusive (Fig. 6). A deeper understanding of the network dynamics of fluctuating bond formation and breakage events opens new doors for the design of inhibitors, or to better understand the impact of point mutations on fertility. It might help to improve on methods of genetic screening that could indicate the compatibility of partners, or potentially might provide a structure-based rationale of fertilization failures. Disturbances to this network, for example, as imposed by conformational changes of the residues heavily involved in the interactions (Fig. 3), or by heavy metals, poisons or drugs that exert the same effect, could prevent fertilization or could be exploited by nature as a regulatory mechanism to block polyspermy. Especially, our simulations of hydrated IZUMO1 with inserted zinc ions allow us to hypothesize a structural mechanism how the well described “zinc spark”^18,19^ could potentially prevent formation of a tight contact between JUNO and IZUMO1 after the first sperm-egg collision (Fig. 6). We also gained structural insights addressing another mystery, namely why JUNO did not show folate binding in solution despite belonging to the folate receptor family (Fig. 5). To validate our methods, in control simulations, we further tested two point mutations, first, TRP148ALA^6,7^ on the JUNO-binding interface on IZUMO1 (Fig. 4), and a second, HIS177GLU^17^ mutation on JUNO associated with fertilization failure in female patients (Fig. 4).

The atomistic insights into the inter-protein bond dynamics revealed that the JUNO-IZUMO1 complex is stabilized by a large network of interactions comprising more than 30 non-covalent bonds and contacts (Fig. 1 and Supplementary Figs. S20–S23 and Movies S1, S2). Upon hydration, IZUMO1 is flattening nearly doubling the contact surface between JUNO and IZUMO1 compared to the crystal structure (Fig. 1). In the simulated time window of 200 ns, the typical bond lifetimes are below 50 ns for the majority of contacts. It was previously suggested that the JUNO-IZUMO1 complex is only transiently present in the fertilization process and that its main role is to create a scaffold for the assembly of larger macromolecular complexes that once formed can initiate the membrane fusion^8,11,40,41^. The large, yet shallow and flexible binding interface, as highlighted in our simulations, makes the JUNO-IZUMO1 complex particularly well suited for this role. The large number of short-lived interactions on an easily accessible surface might ensure a high on-rate, yet allow the complex to potentially be easily broken apart in subsequent stages. Their complexation starts the rapidly evolving fertilization process, despite the presence of disruptive mechanical forces, such as shear and rotational force as generated by the sperm’s tail. The key kinetic challenge is that the JUNO-IZUMO1 contact needs to hold long enough to enable all the local molecular reorganization processes to take place as required to initiate the sperm-egg fusion. Yet, the complex has to break eventually as it might otherwise block fertilization. IZUMO1’s TRP148 seems to play a crucial role, as the TRP148ALA mutation disrupts the JUNO-IZUMO1 binding in vitro^6,7^. Our simulations show now that this mutation progressively reduces the number of interfacial network contacts (Fig. 4, Supplementary Figs. S21, S25 and Movie S4). Our time windows were too short though to simulate the complete unbinding, especially since the numerous neighboring residues formed transient contacts along their dissociation path. Alternatively, if the “boomerang” conformation of IZUMO1 is docked to JUNO, a loosening of the interaction network was again observed showing that IZUMO1’s “straight”, but not the “boomerang” conformation formed stable contacts with JUNO (Figs. 3, S21, S27, S28, and Movies S6, S7). In contrast, JUNO’s HIS177GLU mutation associated with infertility in patients^17^, is positioned away from the binding interface and did not affect the complex’s stability (Figs. 4, S21, S26 and Movie S5).

In the presence of Zn^2+^ ions, or potentially of other heavy metals, IZUMO1 on the surface of an approaching sperm might get conformationally primed to hinder the binding to JUNO. We found that Zn^2+^ ions are able to stabilize the “boomerang” conformation of IZUMO1 (Fig. 2 and Movie S9). Nature might have evolved different layers of security to prevent polyspermy, that could act in subsequent, yet complementary manners. It is well described that Zn^2+^ ions are released in a “zinc spark” within minutes after the first sperm-egg fusion has occurred, which initiates events known to cause a polyspermy block via zona pellucida hardening^18,19^. Moreover, once the Zn^2+^ ions are released, the sperm behavior towards an egg switches from chemoattractive to chemorepulstive, yet the identity of the sperm binding protein of zinc has not been confirmed^20^. Our MD simulations now suggest that the locally released Zn^2+^ interacting with IZUMO1 prior to the JUNO-IZUMO1 complexation could inhibit the process by shifting IZUMO1 towards the “boomerang” conformation. This could be exploited as a rapid, molecular polyspermy block for those sperms that still reach already fertilized egg, but do not adhere to the membrane (Fig. 6). Whether additional JUNO-IZUMO1 complexes that had already formed at the moment of the first sperm fusion with egg membrane could also dissociate as a result of the zinc release remains unknown. While a brief simulation indeed suggests that this might be the case (see Fig. S32), addressing this question by MD is hampered by the computational protocol needed to first manually insert Zn^2+^ ions into IZUMO1, remove steric clashes and then re-equilibrate. This question should thus best be followed up experimentally.

Various other heavy metal ions are known to influence fertility either passively upon environmental exposure^31,32^, or actively by preventing fertilization via intrauterine devices releasing copper ions^43^. The impact of copper ions on IZUMO1’s conformation is inferred here but was not directly simulated. Due to their similarity in charge and size with zinc ions though (Table S3), we speculate that their presence in the fertilization environment could affect IZUMO1 in the similar manner, making it unable to form sufficiently stable transient interactions with JUNO.

Our MD findings might explain another mystery too: despite the existing link between successful conception and folate supplementation^22–24^, JUNO was reported not to bind folate in solution^4,21^, even though it is a folate receptor family member. While in complex with IZUMO1, our MD simulations were able to capture folate binding events to JUNO. In the presence of IZUMO1, the entrance to the putative folate binding pocket remained unobstructed by JUNO’s inhibitory loop and the folate insertion was possible after getting attracted to the binding pocket by the charged residues on JUNO (Figs. 5, S2 and Movies S12, S11). Folate fits inside JUNO’s binding pocket, yet MD suggests that its chances to enter the binding pocket increase if the inhibitory loop is stabilized in an open conformation, as enabled by the presence of IZUMO1 (Fig. 6). In contrast, MD simulations did not reveal any specific folate binding events to JUNO alone (Fig. S4 and Movie S13), where the inhibitory loop at its entrance mostly blocked the entrance to the binding pocket (Fig. S14). This inhibitory loop is one of the most diverse regions of JUNO among different species, in length as well as in primary sequence^6^, and it is much longer than in other FRs^21^. To ask whether the loop tunes the dynamics of the JUNO-IZUMO1 complex, we shortened the loop of human JUNO to the length that is found in mice and found that this partially disrupted the JUNO-IZUMO1 interaction network (Fig. S21). Interestingly, the folate insertion into the binding pocket had no noticeable effect on JUNO-IZUMO1 binding (Figs. S21, S29). Further binding assays of folate insertion into JUNO while bound to IZUMO1 are necessary to establish how folate might regulate molecular aspects of the fertilization process.

Even though fertilization is the first event in the propagation of species, how species-specific recognition and sperm-egg fusion is steered by the molecular complexity of the proteins exposed on the sperm versus egg surfaces remains elusive. Beyond the identification of additional players^8,44–46^, insights into the dynamics of their interactions is prone to reveal crucial information of a process that happens far out of equilibrium. Our findings provide fundamentally new, high-resolution insights into the dynamics of the central molecules that mediate the first specific sperm-egg recognition event. The derived structural hypotheses how bond dynamics steer the early binding events might inspire new experiments how to promote or interfere with fertilization. Future experimental binding assays of folate insertion into JUNO, while bound to IZUMO1, or in vitro fertilization systems with addition of Zn^2+^ ions, are necessary to experimentally test the hypotheses presented here in order to establish how Zn^2+^ ions and folate binding might regulate molecular early aspects of the fertilization process. MD insights offer a larger structural foundation that could be exploited to develop pharmaceutical agents regulating fertilization, for example via control of the conformational state of IZUMO1, or by the enhanced presence of heavy ions, or even suggest new residues that could be targeted to control the binding interface, beyond the once suggested by the crystal structure. As the JUNO-IZUMO1 complex is formed transiently, deciphering the structural mechanisms that define its dynamic stability might inspire the community to evaluate how to better predict or treat infertility, or how to target crucial interactions only seen in the hydrated structure for non-hormonal contraception, or how to improve on in vitro fertilization techniques.

## Methods

### Structure modeling

Since the known interaction interface of the JUNO-IZUMO1 complex is located between their extracellular domains, we focused here on those regions that are available in the Protein Data Bank (PDB): JUNO residues 20–228 and IZUMO1 residues 22–254 (Fig. 1A). Two research groups^6,7^ have published crystal structures of the human JUNO-IZUMO1 complex (PDB IDs: 5F4E, 5JKC, 5JKD, 5JKE). Unbound forms of JUNO are also available for human PDB IDs: 5F4Q^6^, 5JKA^7^, 5JKB^7^, and of IZUMO1 for human- 5F4T^6^, 5F4V^6^, 5JK9^7^.

We were interested in JUNO and IZUMO1 separately, and in complex with each other, which resulted in a total of four main systems: JUNO alone, IZUMO1 alone "straight", IZUMO1 alone "boomerang" and the JUNO-IZUMO1 complex. For uncomplexed IZUMO1, two different starting conformations were considered, namely the “straight”^7^ as well as the “boomerang”^6^, as the respective crystal structures show significant conformational differences. Since the JUNO structure did not present major conformational changes between its crystal structure alone and in the complex with IZUMO1, we have only used the latter (PDB 5JKE). As the loop located at the side of the JUNO’s putative folate-binding pocket had not been resolved in any of the crystal structures (residues 118 to 122), we modeled it here by homology using the SWISS-MODEL server^47^ and verified it in Robetta server that uses the de novo Rosetta method for the prediction of unknown inserts^48^. For IZUMO1 in the “boomerang” conformation, its crystal structure alone was used as a template (PDB 5F4T), while the "straight" IZUMO1 conformation was extracted from the complex (PDB 5JKE). All of the starting protein structures were refined using the SWISS-MODEL server homology modeling pipeline^47,49^, which relies on ProMod3^50^, a comparative modeling engine based on OpenStructure. Finally, the JUNO-IZUMO1 complex was created using the protein models and aligning them based on the crystal structure (PDB 5JKE) with minor distance adjustments to avoid sterical clashes. The resulting structure of the JUNO-IZUMO1 complex (Fig. 1A) used as an input in the complex simulations, contains hydrogens, no gaps in the sequence and correctly captures most of the interactions present in the crystal structures (19 inter-protein hydrogen bonds within 3.5 Å)^6,7^. Due to a slightly different orientation of GLU71 on IZUMO1 associated with the explicit presence of its neighboring residues, two hydrogen bonds described in the crystal structures are not formed (with LYS163 and GLY80 of JUNO^6,7^. Overall, the RMSD over carbon alpha atoms of our systems with respect to the crystal structure (PDB ID 5JKE) when aligned to JUNO is 0.2 Å and 1.1 Å for JUNO and IZUMO1, respectively.

The JUNO-IZUMO1 complex contains three N-glycosylation sites not fully resolved in the crystal structure, i.e. ASN73 on JUNO, and two residues ASN204 and ASN239 on IZUMO1. While we modeled them initially (Fig. S32), our preliminary equilibrium MD simulations showed no change in binding behavior which motivated us to use the deglycosylated complex throughout this study. Further structure modifications (e.g. sterical clashes correction) and mutations were done in QwikMD plugin^51^. Heavy metal binding sites were predicted using online Metal Ion-Binding Site Prediction and Docking Server^33,34^.

### All-atom MD simulations

The structures were then each placed in a cubic box (side length of approximately 160 Å), neutralized and solvated in a physiological NaCl salt concentration of 0.15 M and TIP3 water molecules using QwikMD plugin^51^ in the VMD version 1.9.4a12^52^. All simulations were run with the NAMD version 2.14^53^, using the CHARMM force field^54,55^, and using 2 fs timesteps. The full protocol for each of the runs involved: 2000 steps minimization, stepwise heating between 0 and 310 K, equilibration (with backbone restrains) and production (without any restrains) for 200 ns, unless indicated differently. The pressure of 1 atm was controlled with a Langevin piston. The short range non-bonded interaction cutoff was set to 12 Å with shifting function at 10 Å. The long-range electrostatic interactions were calculated using the Particle-Mesh Ewald method^56^. Each system was simulated in 10 independent runs of 200 ns each (2 *µ*s per system), unless indicated differently. A summary of the completed simulations can be found in Table S1. The simulations were run at the Piz Daint supercomputer from the Swiss National Supercomputing Center in Lugano, Switzerland (CSCS).

### Analysis and visualization

Simulation snapshots and videos were rendered with VMD^52,57^. Non-covalent interactions in our analyses were detected and scored with PyContact^28^ using the default H-bond parameters, i.e., an acceptor to hydrogen distance cutoff of 2.5 Å and an angle cutoff of 120°. Only interactions with the score bigger than 1 were used to define protein-protein interactions and 0.1 for protein-ligand interactions. Clustering was performed using TTClust^58^. The trajectory analyses were done using MDAnalysis^59,60^, adapted to our specific needs using custom python scripts and Jupyter notebook. For details see Supplementary Information S1.

### Supplementary Information

Supplementary Information 1.

Supplementary Information 2.

## Data Availability

The Protein Data Bank (PDB) structures used as modeling input for JUNO-IZUMO1 complex: 5F4E, 5JKC, 5JKD, 5JKE; for unbound JUNO: 5F4Q, 5JKA, 5JKB; and of unbound IZUMO1: 5F4T, 5F4V, 5JK9. MD trajectories can be shared upon request to the corresponding author.
