# Supplementary material for: Molecular dynamics of JUNO-IZUMO1 complexation suggests biologically relevant mechanisms in fertilization
Source: Sci Rep. 2023 Nov 20;13:20342. doi: 10.1038/s41598-023-46835-0 (PMC10663542; doi:10.1038/s41598-023-46835-0)
Supplement: Supplementary file 2 — Supplementary Information 2. [file 41598_2023_46835_MOESM2_ESM.pdf]

Supplementary Information
Molecular Dynamics of JUNO-IZUMO1 complexation suggests biologically relevant mechanisms in fertilization

Paulina Pacak[1], Carleen Kluger[2,3] and Viola Vogel[1*]

[1*]Department of Health Sciences and Technology, ETH Zurich, Zurich, Switzerland.
[2]Lehrstuhl für Angewandte Physik and Center for NanoScience, Ludwig-Maximilians-Universität München, Munich, Germany.
[3]Current address: Evotec München GmbH, Neuried, Germany.

*Corresponding author(s). E-mail(s): viola.vogel@hest.ethz.ch;
Contributing authors: paulina.pacak@hest.ethz.ch;
carleen@klugerlab.de;

Table of contents

Summary of Molecular Dynamics (MD) simulations 2

Detailed results of folate binding to the JUNO-IZUMO1 complex 3

Detailed results of non-specific folate binding to JUNO alone 9

JUNO's loop in proximity to the putative folate binding pocket got stabilized in the open conformation when in complex with IZUMO1 12

Shortening of the inhibitory loop of JUNO showed only minor effects on the JUNO-IZUMO1 binding interface 15

Conformational Analysis 17

Structural clustering 30

Contact area analysis 34

Exemplary timelines of JUNO-IZUMO1 interactions. 35

Heavy ion binding to JUNO and IZUMO1 51

JUNO-IZUMO1$_{boomerang}$ complex with Zn^{2+} ions 54

Glycosylated JUNO-IZUMO1 complex 56

Movie Legends 57

Summary of Molecular Dynamics (MD) simulations

System	Nb of trajectories	Time per trajectory
**IZUMO1**$_{straight}$	10	200 ns
**IZUMO1**$_{boomerang}$	10	200 ns
**IZUMO1 with** Zn^{2+}	10	200 ns
**JUNO**	10	200 ns
**JUNO**$_{shortloop}$	10	200 ns
**JUNO**$_{H177Q}$	10	120 ns
**JUNO-IZUMO1 complex**	10	200 ns
**JUNO**$_{shortloop}$**-IZUMO1 complex**	5	200 ns
**JUNO-IZUMO1 complex with folate away**	10	100 ns
**JUNO-IZUMO1 complex with folate partially bound**	10	20 ns
**JUNO with folate partially bound**	10	20 ns
**JUNO-IZUMO1**$_{boomerang}$ **complex**	10	200 ns
**JUNO**$_{H177Q}$**-IZUMO1 complex**	10	120 ns
**JUNO-IZUMO1**$_{W148A}$ **complex**	10	200 ns

Table S1: ***List of the simulations and their duration time for all trajectories created and assessed in this study.*** *All systems were protonated at physiological pH (~7.4), solvated in explicit TIP3 water molecules and in the presence of 0.15 M NaCl.*

Detailed results of folate binding to the JUNO-IZUMO1 complex

To assess by MD whether folate can bind to JUNO in the presence of IZUMO1 [1, 2], folate and JUNO-IZUMO1 complex were placed in a solvent box. Folate was positioned 50 Å away from the folate binding pocket and was allowed to diffuse freely for 100 ns in 10 independent replicas.

We captured one event where folate approached the complex after 20 ns, recruited by IZUMO1, and bound non-specifically to JUNO's surface and remained there for the rest of the simulation (Figure S3A, run #10). Further, two events occured, where folate approached the complex briefly and in unspecific ways (Figure S3A, runs #3 and #4), and finally one in which the step-wise binding of folate into the central pocket could be observed within 45 ns. Once inserted into the pocket, the ligand position was stable for the remaining time, with the inhibitory loop partially blocking the free exit route (Movie S12). The folate in the observed pose was only partially embedded inside of JUNO, not reaching as deep inside the pocket as it was reported for the FRα [3] (Figure S2D). Its conformation was more bent, supporting previous MD studies of FR ligands (folate and analogues [4, 5] and the pose was similar to the one reported in the computational study of FRα [5].

The intermediate binding state (from t = 43 ns of previously successful simulation) was used as a starting structure to test possibility of a deeper insertion of folate into the binding pocket. Simulations were repeated 10 times for 20 ns for the JUNO-IZUMO1 complex, referred here as "refined" binding. The binding of folate to JUNO alone was also tested (see details in next section), to ask whether the computationally observed binding is unique to JUNO's complexation with IZUMO1, or might be due to the strong hydrophobicity of the putative folate binding pocket.

In our "refined" simulations of the folate binding to the JUNO-IZUMO1 complex, we have observed two cases, where folate inserted to the binding pocket. In first case, the shallow pose was similar to the one seen in the spontaneous binding (Figure S3B, run #8). In the second, folate insertion reached the same depth as in the known FRα structure [3] (Figure S2D and Movie S11). In 5 other simulations, folate bound briefly in a non-specific way to either JJUNO, IZUMO or to their interface (Figure S3B, run #1, run #2, run #3, run #5, run #7). Interestingly, in all successful pocket-binding events, JUNO's residues ARG154 and GLY156 were involved in mediating the first JUNO-folate contact, subsequently guiding folate's way to the central binding pocket. At most eight hydrogen bonds, two salt bridges, and two π-stacking interactions were observed.

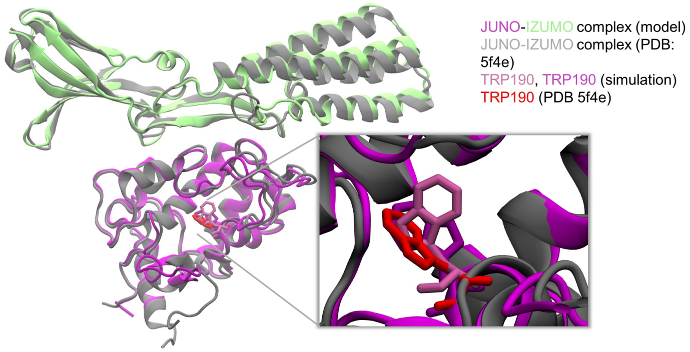

Fig. S1: ***Various conformations of TRP190*** *in the simulations of the hydrated JUNO-IZUMO1 complex. In the course of the MD simulations TRP190 rotates enlarging the accessible volume of the folate binding pocket.*

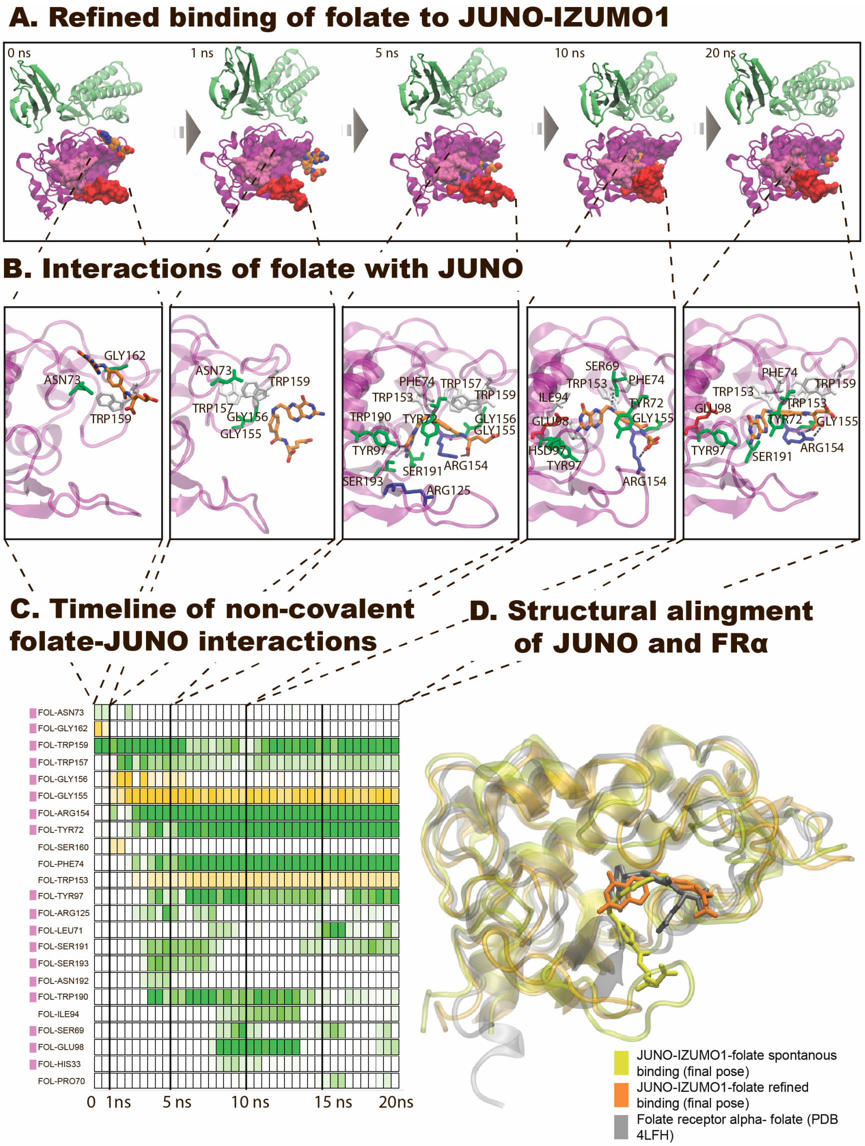

Fig. S2: ***In silico refined folate binding to JUNO in complex with IZUMO1.*** *(Caption on the following page.)*

Fig. S2: ***In silico refined folate binding to JUNO in complex with IZUMO1***. *A. Representative snapshots of the successful folate binding during 20 ns equilibrium MD simulation starting from partially bound state (run #4). Proteins are shown in the cartoon representation (purple: JUNO, green: IZUMO1), residues around the central binding pocket of JUNO are shown as surface representation (red: residues 110 to 125, pink: 29 to 35, purple: pocket residues). The first frame is extracted from the partially bound state of the previously successful simulation and equilibrated, followed by the production simulation. ARG154 and GLY156 seem to play an essential roles in promoting folate to slide into the pocket. Deeper insertion is achieved and the final pose is closer to the known folate binding orientation inside the folate receptor (see panel D). B. Magnified view of the main folate-JUNO interactions in the corresponding snapshots. Protein residues are colored based on their type (green: polar, red: acidic, blue: basic, white: non-polar). C. Timeline of non-covalent interactions between folate (FOL) and JUNO in the refined run. Each box represents equal time segment. Pink labels mark hydrogen bonds. The interaction scores are coded by color intensity and color-coded based on the nature of the interaction (green: side chain interaction; yellow-backbone interaction (made with PyContact [6]). D. Alignment of the last frames of the successful folate-JUNO-IZUMO1 binding simulations (fully spontaneous and refined) to the known crystal structure of the folate bound to the folate receptor alpha (PDB: 4LRH).*

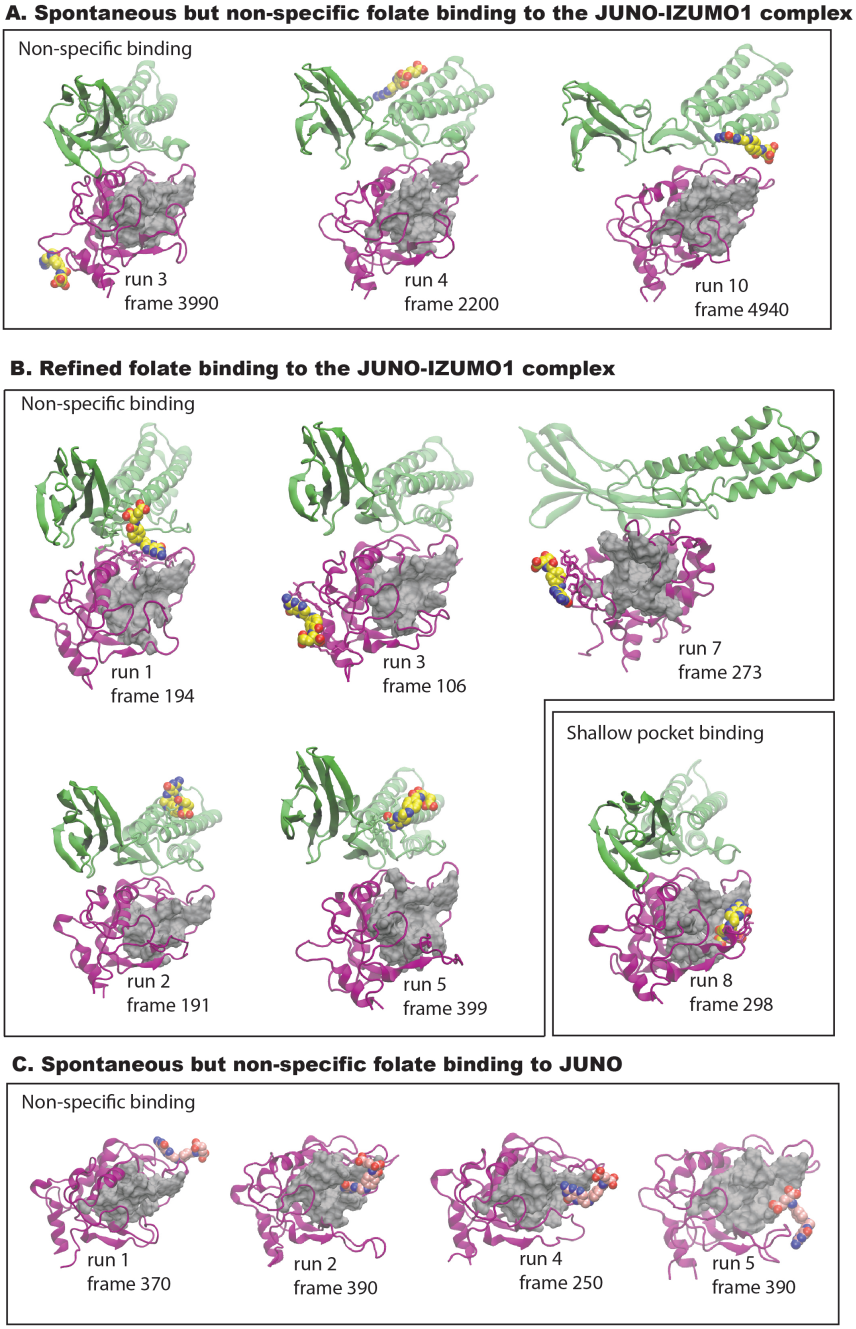

Fig. S3: ***Non-specific folate binding to JUNO in silico under different conditions.*** *(Caption on the following page.)*

Fig. S3: ***Non-specific folate binding to JUNO in silico under different conditions***. *A. Representative snapshots of the partial folate binding to the JUNO-IZUMO1 complex during a 100 ns equilibrium MD simulations starting from the unbound state. B. Representative snapshots of the non-specific folate binding to the JUNO-IZUMO1 complex during a 20 ns MD simulation starting from the partially bound state. C. Representative snapshots of the non-specific folate binding to JUNO alone starting from the partially bound state, whereby folate is not seen to insert into the pocket within the 20 ns of the simulation. Proteins are shown in the cartoon representation (purple: JUNO, green: IZUMO1), residues around the central binding pocket of JUNO are shown as surface representation (red: residues 110 to 125, pink: 29 to 35, purple: pocket residues).*

Detailed results of non-specific folate binding to JUNO alone

In the “refined” simulations, the equilibrated structure of JUNO alone was used. The relative folate and JUNO orientations were used as for the “refined” complex simulations (from t = 43 ns of previously successful simulation) and the slight adjustments to eliminate the sterical clashes were made. The system was than solvated and simulations were run in 5 repeats.

In simulations of folate with JUNO alone, folate slipping into the pocket was not observed, however, in 4 out of 5 runs folate unsuccessfully screened the side of it (Figure S3). In run #4, even though the first interaction with TRP159 is formed, and later enhanced by GLY156 and TRP157 as in case of successful binding to JUNO in the complex with IZUMO1, the inability to form a hydrogen bond with residue ARG154 makes the entrance to the pocket impossible and folate ultimately unbound from the surface (Figure S4 and Movie S13). This is partially due to the reorganization of the pocket residues, that engaged JUNO’s ARG154 forming additional H-bond interactions with the inhibitory loop residues: ALA118, PRO119 and SER120. This effectively blocked the loop in the closed conformation. Taken together, those results suggest that the stabilization of the inhibitory loop and the putative folate binding pocket is only possible in the presence of IZUMO1. For FRα in a comparable, yet more complex setup containing the lipid membrane [5], spontaneous folate binding was observed in all of their 4 repeated simulations within the 240 ns simulation time. Even though shorter simulation times were tested here, this could suggest that the attraction forces in FRα are more prominent than in JUNO, or that the presence of the membrane further promotes the binding.

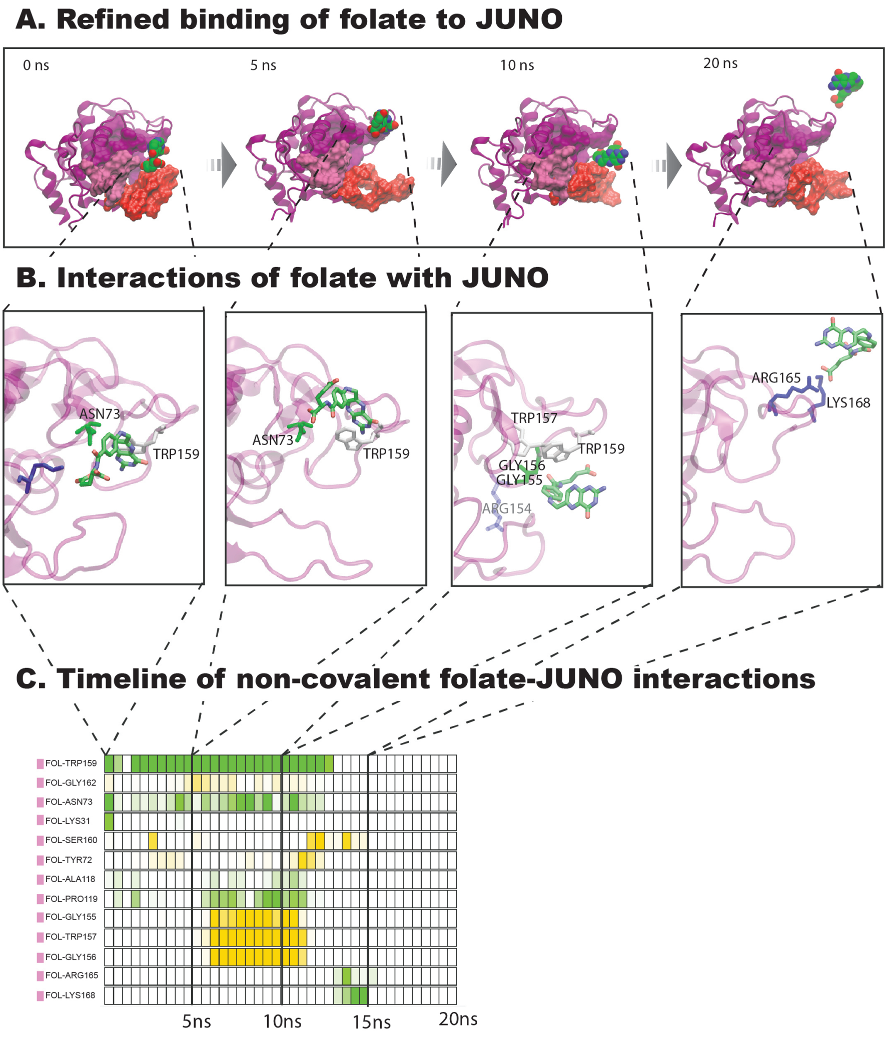

Fig. S4: ***Non-specific folate binding to JUNO alone in silico.*** *(Caption on the following page.)*

Fig. S4: ***Non-specific folate binding to JUNO alone in silico.*** *A. Representative snapshots of non-specific folate binding in 20 ns equilibrium MD simulation starting from partially bound state (run #4). Proteins are shown in the cartoon representation (purple: JUNO, green: IZUMO1), residues around the central binding pocket of JUNO are shown as surface representation (red: residues 110 to 125, pink: 29 to 35, purple: pocket residues). The first frame is extracted from the partially bound state of the previously successful simulation and equilibrated. B. Magnified view of of the main folate-JUNO interactions in the corresponding snapshots. Protein residues are colored based on their type (green: polar, red: acidic, blue: basic, white: non-polar). C. Timeline of non-covalent interactions between folate (FOL) and JUNO alone in the refined run. Each box represents equal time segment. Pink labels refer to hydrogen bonds. The interaction scores are coded by color intensity and color-coded based on the nature of the interaction (green: folate-side chain interaction; yellow: folate-backbone interaction (made with PyContact [6]).*

JUNO's loop in proximity to the putative folate binding pocket got stabilized in the open conformation when in complex with IZUMO1

JUNO is also a particular member of folate receptors (FR) family, that does not show folate binding activity *in vitro* [1, 2], yet contains large putative folate binding pocket, placed opposite of the IZUMO1 binding interface and partially buried by the long inhibitory loop on its side. As we hypothesize that access to the folate binding pocket might be enabled by structural rearrangements caused by IZUMO1 binding, additional 10 independent simulations of 200 ns each were performed for JUNO alone in addition to the previously mentioned simulations of the complex, and compared between those two conditions. The exemplary trajectories, highlighting the global behavior of JUNO can be seen in Movie S10. During the simulations, JUNO alone in solution maintained its overall secondary structure and only the unstructured loop regions showed some flexibility (Figures S14A and S15A). In particular, its inhibitory loop (FR family-specific inhibitory loop [8] of JUNO between residues 117 and 123) underwent major adjustments on the side of the putative folate binding pocket (Figure S11). JUNO in complex with IZUMO1 generally showed less local movement (Figure S14B and S15B), as many of its loops were at least partially involved in the interaction with IZUMO1 and therefore got stabilized. In many simulations, however, the inhibitory loop underwent major variations and waving/opening motions, even more prominent than for JUNO alone (Figure S11).

In order to quantify, whether the observed variations in the inhibitory loop behavior can be attributed to the complexation of JUNO with IZUMO1, we have performed structural clustering of JUNO, as described in detail below, on all frames from simulations of JUNO alone and in complex with IZUMO1. Structures representing different clusters were most diverse in the inhibitory loop region (Figure S18). We have grouped observed clusters as either "open" (clusters #1 and #3), where access to the pocket is free, "closed" (clusters #4,#5,#7 and #8), where the pocket is fully blocked or collapsed, and "partial" (clusters #2,#6 and 9), where the access to the binding pocket is limited, but still possible (Figure S5). These results showed that the presence of IZUMO1 promoted JUNO's inhibitory loop opening and made its putative folate binding pocket accessible.

In both simulation sets, for JUNO alone and in complex with IZUMO1, the putative folate binding pocket shows high variations in volume that can mainly be attributed to the fluctuations of the inhibitory loop. This observation agrees with the behavior of FRα in a MD study on a cancer-like membrane that found that the volume of the folate binding pocket fluctuates in time [7]. This would suggest that frequent changes in JUNO's pocket volume are not unusual and both of the previously observed binding poses (Figure S2D) could be physiologically relevant. We have also observed a reorientation of JUNO's residues 70 to 75. This loop is connected to the IZUMO1 binding interface

and shifted less frequently when JUNO was bound to IZUMO1. This suggests that IZUMO1 binding might stabilize the binding pocket and could thus allosterically enhance folate binding. Moreover, the observed changes show the dependency between the IZUMO1 binding interface and the position of the long inhibitory loop of JUNO. To verify, whether the inverse effect can be observed, namely if the lack of the long loop can destabilize the binding interface, we have constructed JUNO with a shorter loop to compare its behavior and ability to bind IZUMO1.

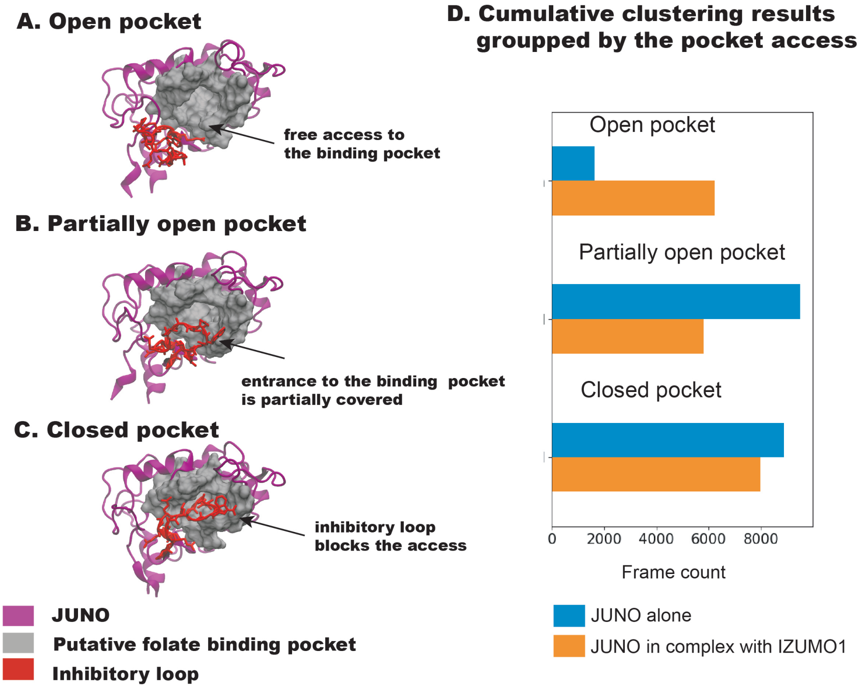

Fig. S5: ***Fluctuations of JUNO's inhibitory loop position.*** *Results from visual inspection, supported by structural clustering (for details see below) allowed us to distinguish three different states of JUNO (A-C), where most variations appeared in the position of the inhibitory loop around the entrance to the putative folate binding pocket. To quantify whether the observed states depend on the presence of IZUMO1, we calculated the number of frames in each state for both of the simulated conditions. D. Results suggest that the open conformation is favored by the presence of its binding partner IZUMO1.*

Shortening of the inhibitory loop of JUNO showed only minor effects on the JUNO-IZUMO1 binding interface

When bound to IZUMO1, the inhibitory loop of JUNO was preferentially found in an open state. To test whether the inverse effect of this relation can be observed, we have created a second model of human-based JUNO by removing amino acids 119-124 that are absent in the sequence of folate receptor alpha (FRα). The inhibitory loop of JUNO is not in direct contact with the IZUMO1 binding interface which led to us to hypothesize that this long loop is allosterically involved in IZUMO1 binding.

We repeated 5 runs simulations of the JUNO construct (JUNO short loop) in the solution alone and in complex with IZUMO1. The structure showed much lower variability, supporting the conclusion that the long inhibitory loop is responsible for the majority of fluctuations in JUNO (Figures S12 and S16)), as the binding interface of the JUNO-IZUMO1 complex with the short loop underwent only minor changes (Figure S24 and Movie S3). The interactions between JUNO's LEU81 and IZUMO1's MET75, VAL77, HIS157 and TRP148 are shortened in their lifetime or not formed at all S6. This suggests that the longer inhibitory loop of JUNO, when compared to FRs or JUNO of other species plays a functional role in the human WT complex functionality, however more studies are necessary to fully understand this effect.

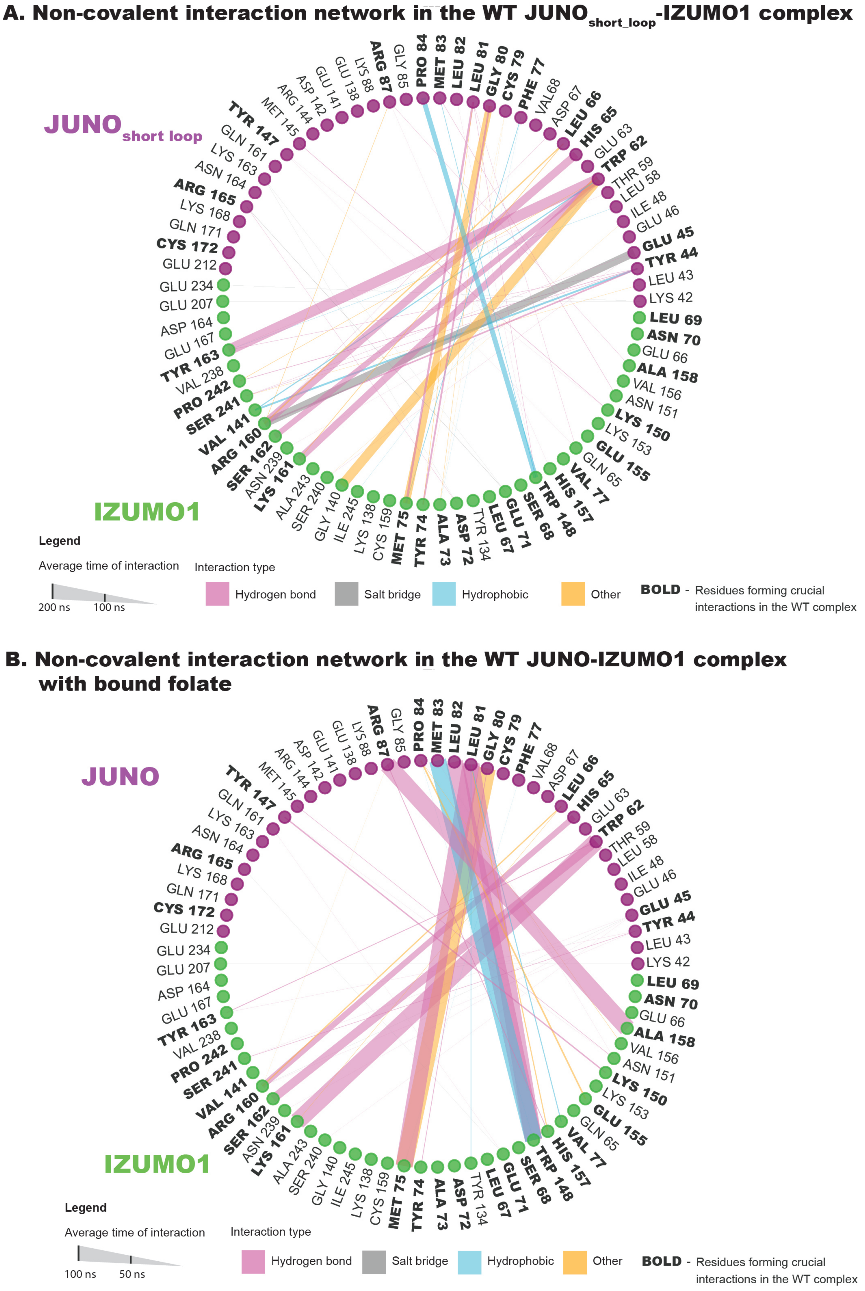

Fig. S6: ***Non-covalent interaction networks.*** *A. Simulations of the complex involving JUNO with the short loop (5 runs). The JUNO-IZUMO1 interaction interface seems partially distorted and the interactions formed are shorter than in the WT. B. Interface analysis between JUNO and IZUMO1 as extracted from the 100 ns simulation of the spontaneous folate binding (1 run).*

Conformational Analysis

Equilibrium Molecular Dynamics (MD) simulations of JUNO, IZUMO1 and their complex provide an ensemble of states that proteins can adapt in given conditions. We used an all-atom, explicit water approach where the proteins are placed in explicit solution and sampled over time of 200 ns in 10 repeats in order to account for the stochastic nature of the used algorithms. Trajectories are than either compared between them in time series or merged to obtain averaged properties and filter out unnecessary noise. Overall conformational variations of JUNO and IZUMO1 proteins alone and in the complex with each other are evaluated by comparative study of the Radius of Gyration, time series of Root Mean Square Deviation (RMSD) and average Root Mean Square Fluctuations (RMSF) to assess local variations.

Radius of gyration

To estimate the shape of the proteins, the radius of gyration (R_g) can be analyzed as an indicator of protein compactness. It was calculated as:

$$R_g = \left(\frac{\sum_i m_i (r_i - r_c)^2}{\sum_i m_i} \right)^{1/2}$$

where m_i and r_i are mass and position of atom i, and r_c center of mass (COM) of a molecule.

IZUMO1 is a rod shaped molecule, so its R_g is relatively high (Figure S7). with the average value over total simulation time of 27.1 Å for the complex, 27.3 Å for IZUMO1 in "straight", and 27.4 Å for IZUMO1 in "boomerang" conformation, showing rather similar overall behavior. The biggest shift is seen in run #7 of the IZUMO1 "straight" simulations, where the *in silico* radius of gyration diminished to less than 24 Å and is associated with spontaneous IZUMO1 bending into the extreme "boomerang" conformation (the angle between the domains of less than 110°). Experimental R_{gyr} values were previously reported for IZUMO1 from SAXS experiments [9], where the ensemble average of SAXS profiles is taken into account [10]. The values obtained using experimental and computational methods are thus not directly comparable. Also the solvent layer around the protein contributes to the SAXS measurements [11]. Nevertheless, looking at the amplitude of the differences observed between IZUMO1 alone and in the complex, provides a hint towards explaining the differences. Experimentally, a change in R_{gyr} of 0.5 Å is observed between the "boomerang" (28.4 Å) and the complex (28.9 Å) conformations [9]. In our equilibrated starting structures the same amplitude of change can be observed between IZUMO1 "straight" alone (27.0 Å) and IZUMO1 "straight" in the complex (27.4 Å), while the "boomerang" structure reaches smaller value (25.9 Å). During the simulation time, the change in R_{gyr} is often higher than 1 Å. Altogether, this shows that our data are not in direct contradiction with the proposed behavior of IZUMO1, but rather could provide additional insights into the conformational transitions of IZUMO1 in solution.

To evaluate shape-related changes of JUNO, we analyzed the R_{gyr} over time for 10 trajectories for both JUNO alone and JUNO in complex with IZUMO1 (Figure S8). Temporary deviations from the crystal structure R_g are seen (16.8 Å), mostly in the higher range, suggesting an elongation of the JUNO structure. For JUNO alone, the majority of trajectories fits within the average value of 16.9 Å. One run though (run #1) showed a second small distribution plateau with the average of 17.4 Å. Visual inspection confirmed that this shift corresponds to the spontaneous inhibitory loop opening motion, rather rare for JUNO alone, and not any other spontaneous conformational changes. This resulted in a partial shape elongation. For JUNO in complex with IZUMO1, larger values are observed and the distribution histogram showed a larger distribution with an average value around 17.1 Å, suggesting that more frequent loop openings cause the shape fluctuations.

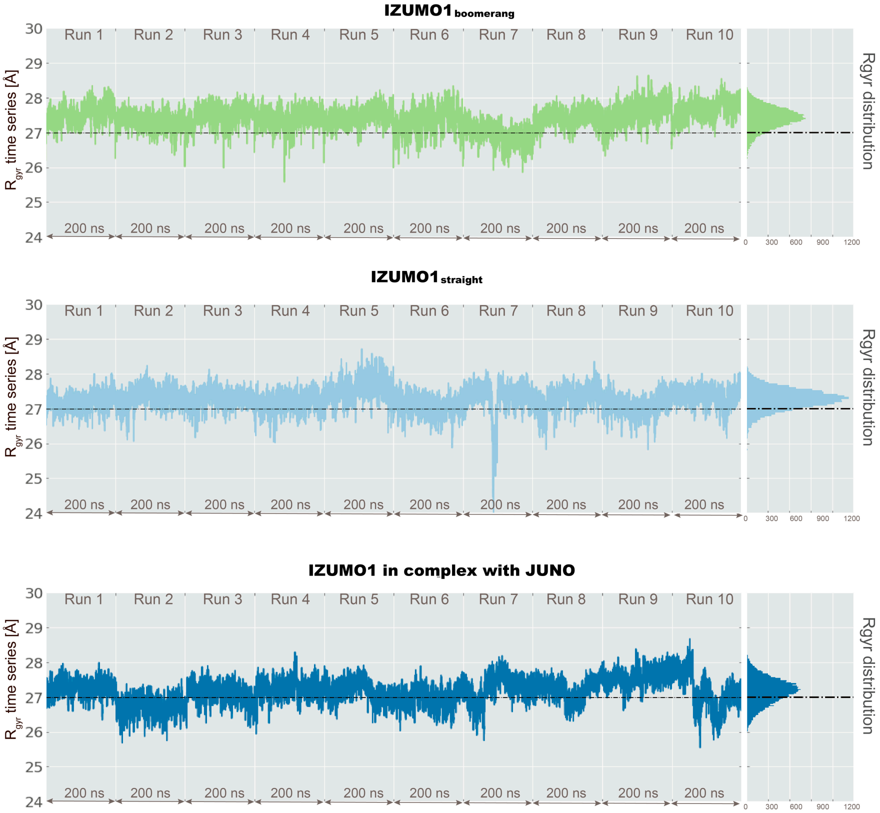

Fig. S7: ***Time series of Radius of Gyration*** *for 10 x 200 ns of MD equilibrium runs for IZUMO1 starting from following states: "boomerang", "straight" and IZUMO1 bound to JUNO. Variations of the radius of gyration reflect the degree of compactness and frequent large changes correspond to the bending motion of IZUMO1 domains and the transition between "boomerang" and "straight" conformations.*

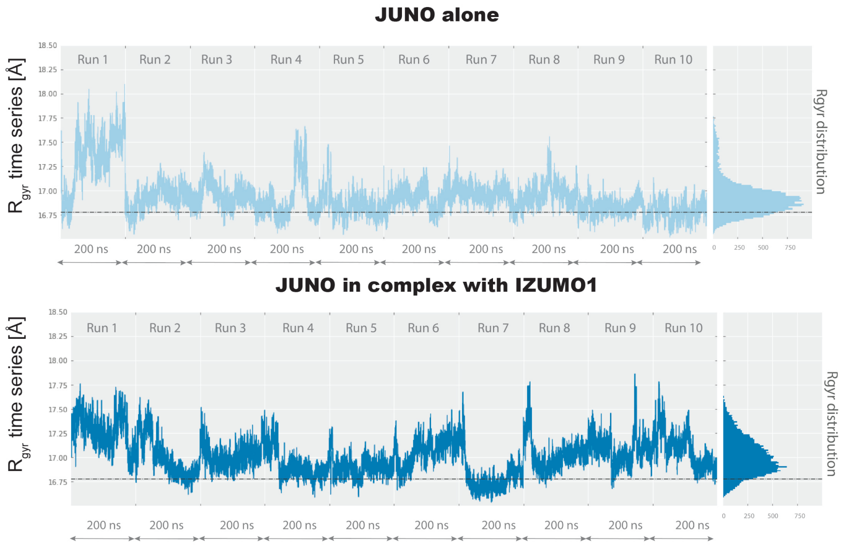

Fig. S8: ***Time series of Radius of Gyration*** *for 10 x 200 ns of MD equilibrium runs for JUNO alone (upper) and JUNO bound to IZUMO1 (lower). The black line indicates the value calculated for the starting (crystal) structure.*

Root Mean Square Deviation (RMSD)

The RMSD with the equilibrated crystal structure as reference was calculated as:

$$RMSD = \left(\frac{1}{N} \sum_i |x_i - x_i^{ref}|^2 \right)^{\frac{1}{2}}$$

where x_i and x_i^{ref} are positions of atom i at the measured state and in the reference respectively, and N is number of atoms in the a molecule.

For IZUMO1, RMSD tracked over the full simulation time on the superposed Cα for all three simulated conditions (Figure S9) confirms that the magnitude of overall conformational changes were smaller in the complex, with an average value of 2.8 Å than in IZUMO1 "straight" (3.2 Å) or IZUMO1 "boomerang" (4.6 Å). Few trajectories showed major RMSD shifts, i.e. for IZUMO1 "straight" (runs #5 and #7) and in the complex (runs #7, #8 and #9). Interestingly, while those shifts in IZUMO1 alone seem to be mostly associated with bending motions, the changes in the complex were related to the position of residues 67-71, that can adapt either extended conformation, creating additional contacts with JUNO, or partially bent conformation interacting with IZUMO1's helical bundle.

Comparison of the RMSDs from all the trajectories of JUNO alone and in complex can be found in Figure S10. In the first few nanoseconds in each run, the protein undergoes adjustments related to structure relaxations, and this period is longer for JUNO in complex due to local rearrangements caused by IZUMO1 binding. The overall structure of hydrated JUNO was rather stable with an average RMSD over Cα of 3.2 Å for simulations alone (Figure S10A) and RMSD of 3.7 Å for JUNO bound to IZUMO1 (Figure S10B). A dual RMSD distribution of all trajectories is seen though, centering around values of 2.9 Å (for initial part of each run) and 4 Å (in later stages of each run). More detailed analyses of the RMSDs, separating JUNO into segments based on its secondary structural features (Figure S11), can attribute the magnitude of the structural changes to different regions of the protein. The inhibitory loop modeled from scratch on the side of the putative folate-binding pocket is the most flexible region displaying higher RMSD values and a wider distribution. The flexible inhibitory loop stabilized more often in the presence of IZUMO1.

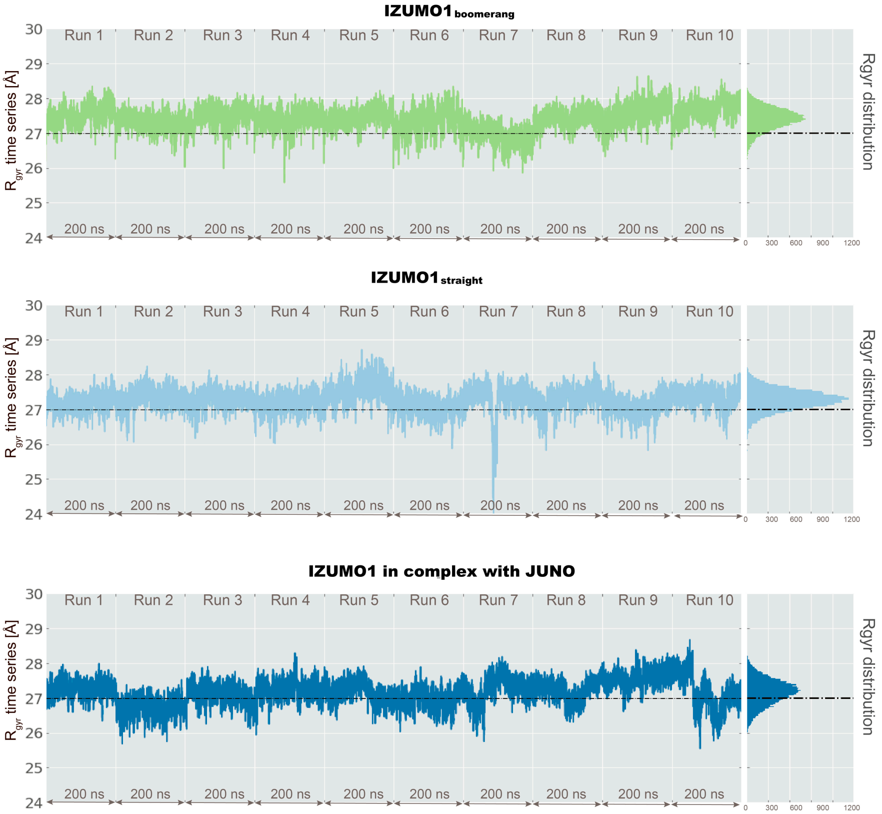

Fig. S9: ***Time series of RMSD*** *for 10 x 200 ns of MD equilibrium runs for IZUMO1 in the "boomerang" or "straight" conformation and for IZUMO1 bound to JUNO.*

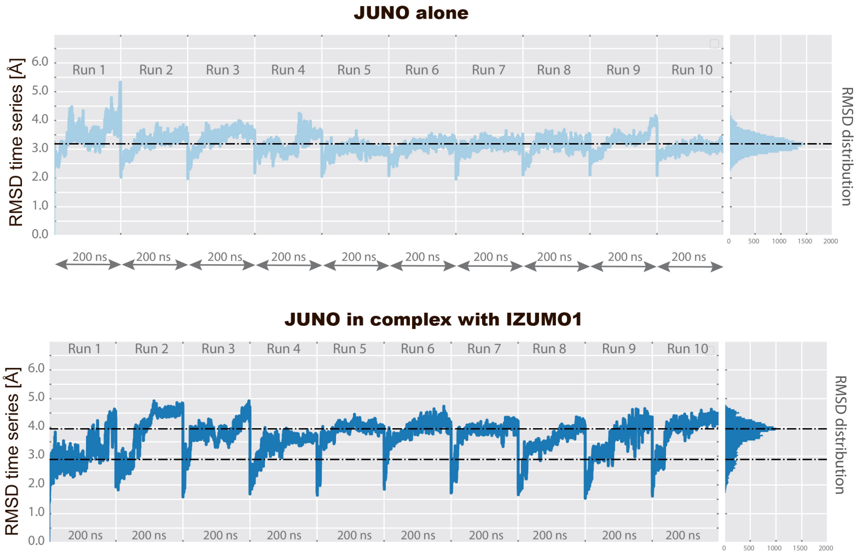

Fig. S10: ***Time series of RMSD*** *for 10 x 200 ns MD of equilibrium runs over Cα of JUNO alone in solution (upper) and JUNO bound to IZUMO1 in solution (lower). JUNO alone showed minor variations centering around the RMSD of 3.2 Å. In the complex a longer adjustment period was observed, that gave rise to the dual RMSD distribution centering around 2.9 Å (for initial part of each run) and 4 Å (in later stages of each run). Each frame was aligned with the energy minimized structure from the first run.*

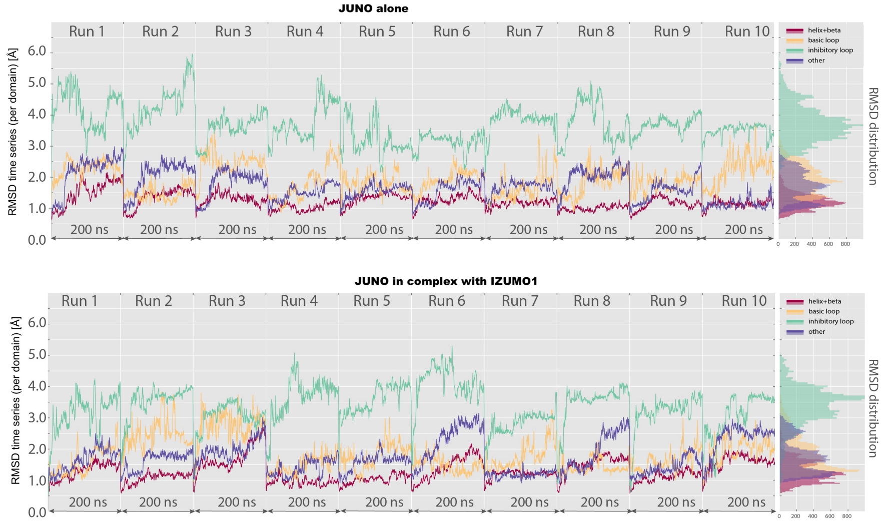

Fig. S11: ***Time series of RMSD for different domains of JUNO*** *for 10 x 200 ns of MD equilibrium runs for JUNO alone in solution (upper) and JUNO bound to IZUMO1 in solution (lower).*

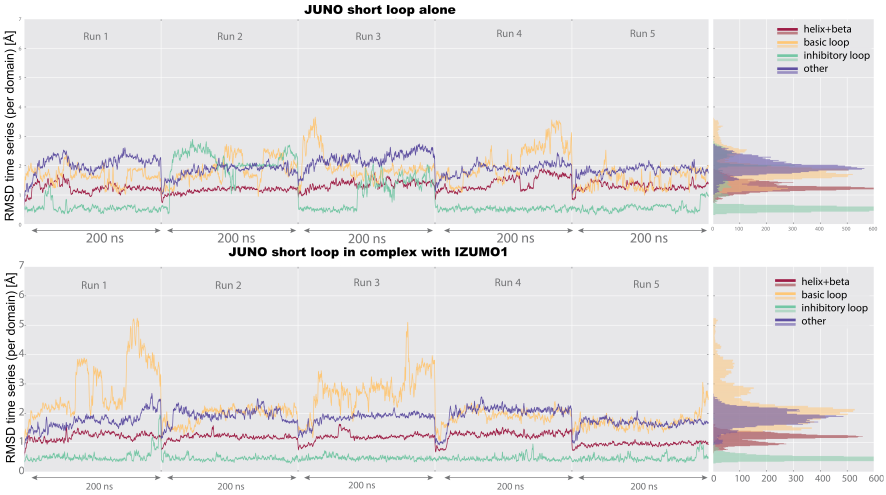

Fig. S12: ***Time series of RMSD*** *for different domains of JUNO with shortened loop (without residues 119-124) for simulation alone (upper) and bound to IZUMO1 (lower).*

Root Mean Square Fluctuations (RMSF)

To assess local changes in protein structure across different simulations, the RMSF of the carbons alpha was projected on the three dimensional structure of each system. Generally, IZUMO1 (Figure S13) was less dynamic when bound to JUNO, than when alone, and its hinge region, where the majority of inter-protein contacts are located was less susceptible to fluctuations, as expected. Between the two starting structures of the simulations IZUMO1 alone, either being "straight" or in the "boomerang" conformation, the latter showed higher RMSF values on both of its termini. This distribution of highly flexible regions across IZUMO1 suggests that the flexibility of IZUMO1 originates from the hinge bending motions.

Overview of the RMSF of the Cα projected on the structure of JUNO (Figure S14) was used to pinpoint its most change-prone regions. It showed mostly low fluctuations with only the inhibitory loop in the simulations of the complex reaching higher values (11.5 Å) due to its opening. These results also confirmed the stabilization of the secondary structure in JUNO's helix and beta sheet regions and confirmed the variability of the residues in the unstructured, loop regions. Interestingly, one region in particular, located opposite of the inhibitory loop at residues GLU212, PRO213, ALA214, GLN215 (Figure S14), presented higher fluctuations than the rest of the protein, even in the presence of IZUMO1. It could be caused by the accumulation of hydrophobic, yet solvent-exposed residues in its proximity.

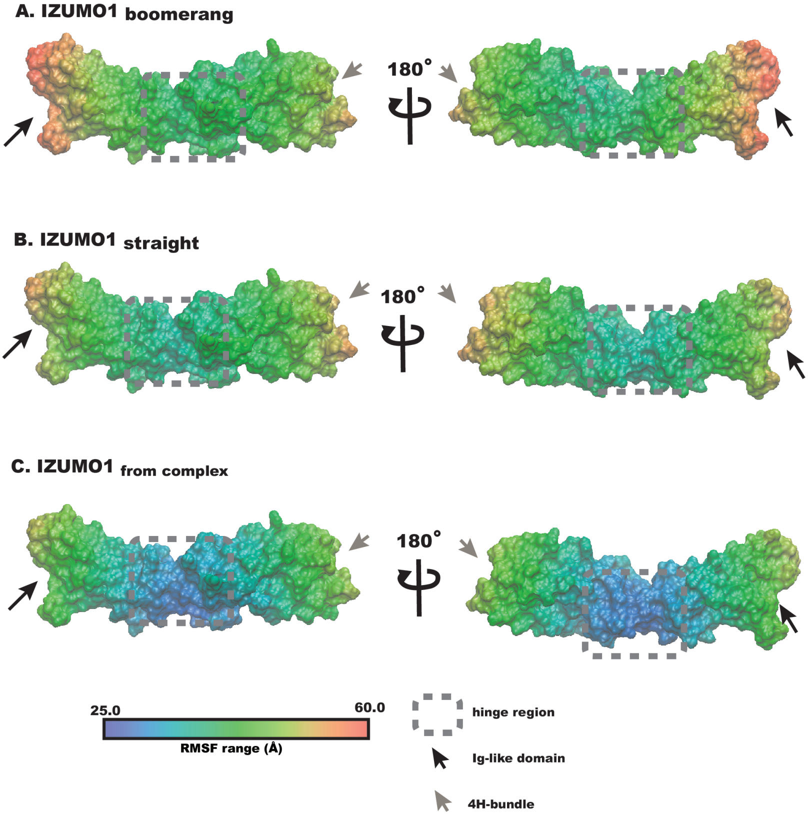

Fig. S13: ***Average RMSF (over 10 x 200 ns) projected on the 3D surface representation of equilibrated IZUMO1.*** *Results from the simulations of the IZUMO1 A. "straight", B. "boomerang" and C. in the complex with JUNO (not shown). In simulations of IZUMO 1 alone there was little difference between "straight" and "boomerang" conformations, while its terminal regions showed most of the fluctuations. IZUMO1 consistently showed bending motions. In the simulations in the presence of JUNO, strong stabilization of the hinge region was observed.*

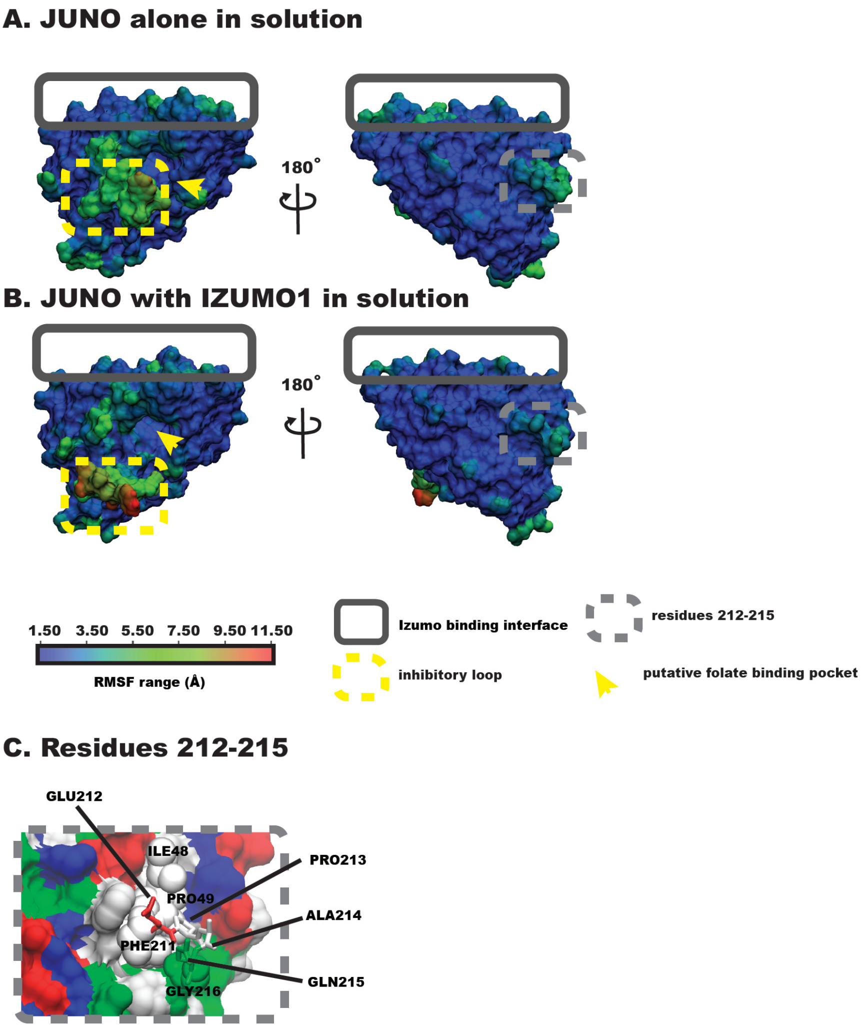

Fig. S14: ***Average RMSF (over 10 x 200 ns) projected on the 3D surface representation*** *of A. equilibrated JUNO alone and B. JUNO in the presence of IZUMO1 (not shown). Highly flexible regions often mark additional functions of the protein and the inhibitory loop (yellow dashed line) on the side of the folate binding pocket (yellow arrow) is the most flexible one (red and green coloring). C. Zoom-in of the flexible residues 212-215 (sticks representation) and the surrounding hydrophobic residues (spheres). They could be involved in the membrane binding or in dimerization. Residues are colored according to their type: red-acidic, blue-basic, green-polar, white-non polar.*

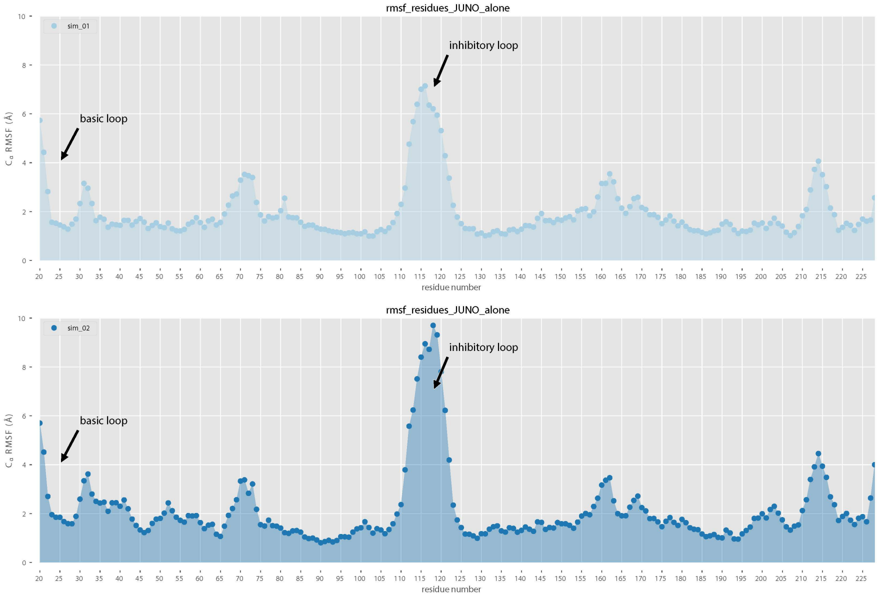

Fig. S15: *RMSF per residue for JUNO alone (upper) and in a complex with IZUMO1 (lower).*

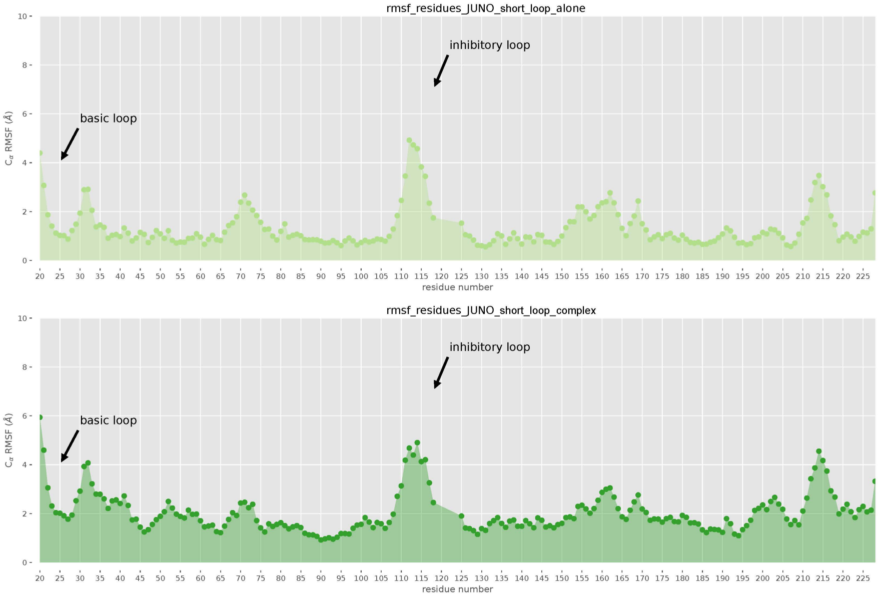

Fig. S16: *RMSF per residue for JUNO with the shortened loop (removed residues 119-124) in simulations alone (upper) and in a complex with IZUMO1 (lower).*

Structural clustering

To compare ensembles of structures or states, RMSD- based structural clustering of protein conformations was performed using TTClust [12] according to the procedure established in [12] with ward clustering method of highest variance. For IZUMO1 or JUNO alone and in the complex with each other, all the frames available from MD simulations (3 conditions x 10 x 200 ns for IZUMO1 and 2 conditions x 10 x 200 ns for JUNO) were assessed. The elbow algorithm suggested three major conformational states for IZUMO1 and four major conformational groups for JUNO (Figure S19), however, to reduce intra-cluster variability in case of the intermediate states for the inhibitory loop positions, nine further clusters were analyzed (Figure S18), to gain a better overview of intermediate structures.

For IZUMO1, our main goal was to verify the bias imposed by single value threshold between "straight" and "boomerang" conformation. The results are shown on Figure S17. Those analysis identified three major clusters with representative states corresponding roughly to IZUMO1 "boomerang" (Figure S17, D, red), IZUMO1 "straight" (Figure S17, D, green) and an intermediate state between them (Figure S17, D, blue). Only 11.9% of frames were classified as "boomerang" spreading through all the simulations (Figure S17, E), majority of frames has been assigned into the "straight" conformation (53.2%) and the remaining part was classified as intermediate. This suggests that the single threshold angle-based separation overestimated the frames belonging to the "straight" conformation.

Further, the intermediate states identified for JUNO's loop are shown in Figure S18. Nine main clusters were identified, that could be regarded as representative intermediate states of the solvent- influenced dynamics of JUNO. The biggest changes, as predicted, were observed in the inhibitory loop region and around the entrance to the putative folate binding pocket, hence those crucial resides are highlighted on the representative frames (Figure S18 F). Among those intermediate states, we can distinguish between conformations where the pocket is freely accessible (clusters: #1, #3), partially accessible (clusters #2, #6 and #9) and fully closed (clusters: #4, #5, #7, #8). A rather uniform distribution of frames is observed among all clusters, with the exception of cluster #2, gathering the most frames, and cluster #9, with the least members (Figure S18A). Comparison of the most populated clusters for frames coming from the simulations of JUNO alone and JUNO in the complex with IZUMO1, shows visible differences in preferred states (Figure S18D,E). Cluster #7 is a conformation present only in simulations of JUNO alone, and cluster #9 for the JUNO in the complex with IZUMO1. Moreover, the clusters identified as representing a "freely accessible" pocket (clusters #1 and #3) are dominantly populated by frames from JUNO-IZUMO1 complex simulations.

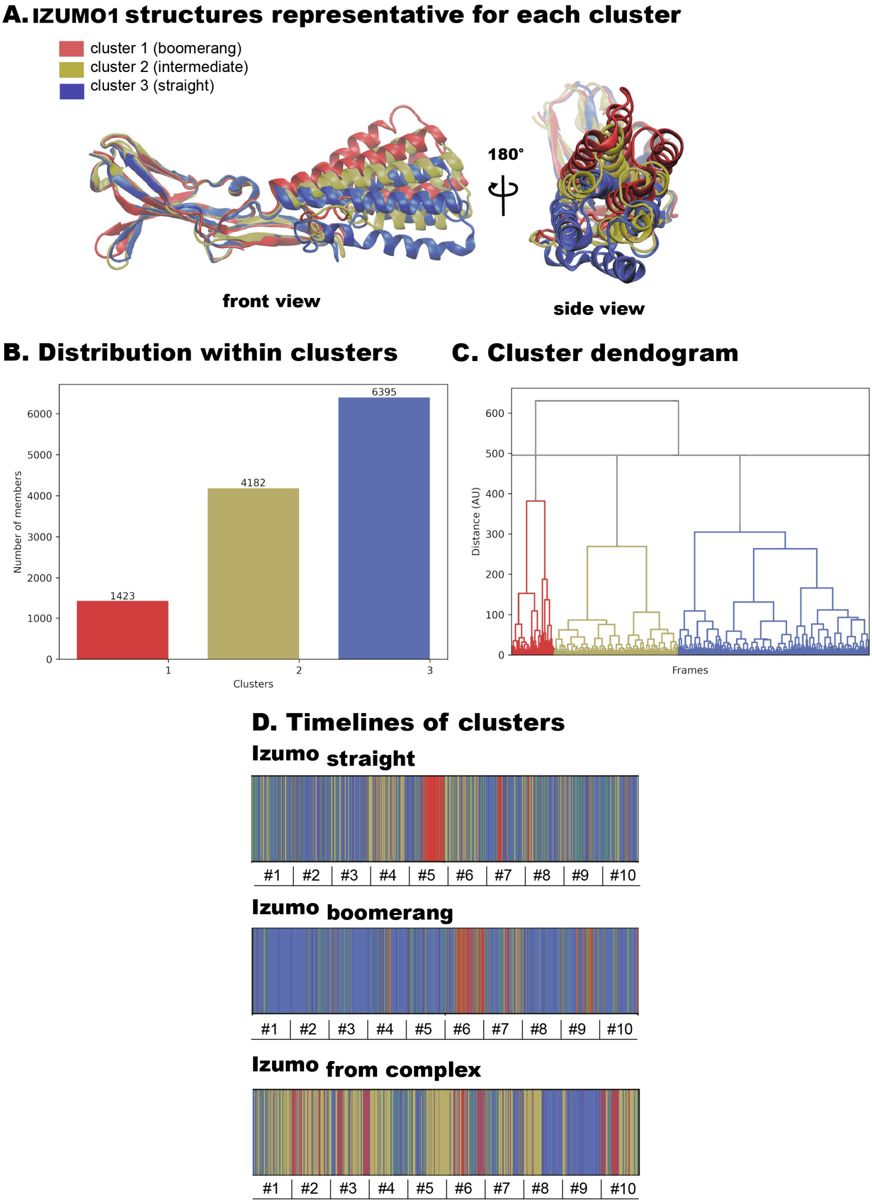

Fig. S17: ***Clustering of IZUMO1's transient structures.*** *A. Three frames showing representative structure for each cluster have three different hinge angles. The clustering was done on trajectories of IZUMO1 "boomerang", "straight" and from the complex with JUNO (4000 frames each set). B,C. Majority of frames has been classified as "straight". D. Timelines represent all 10 runs in consecutive order. Clustering done using TTClust [12].*

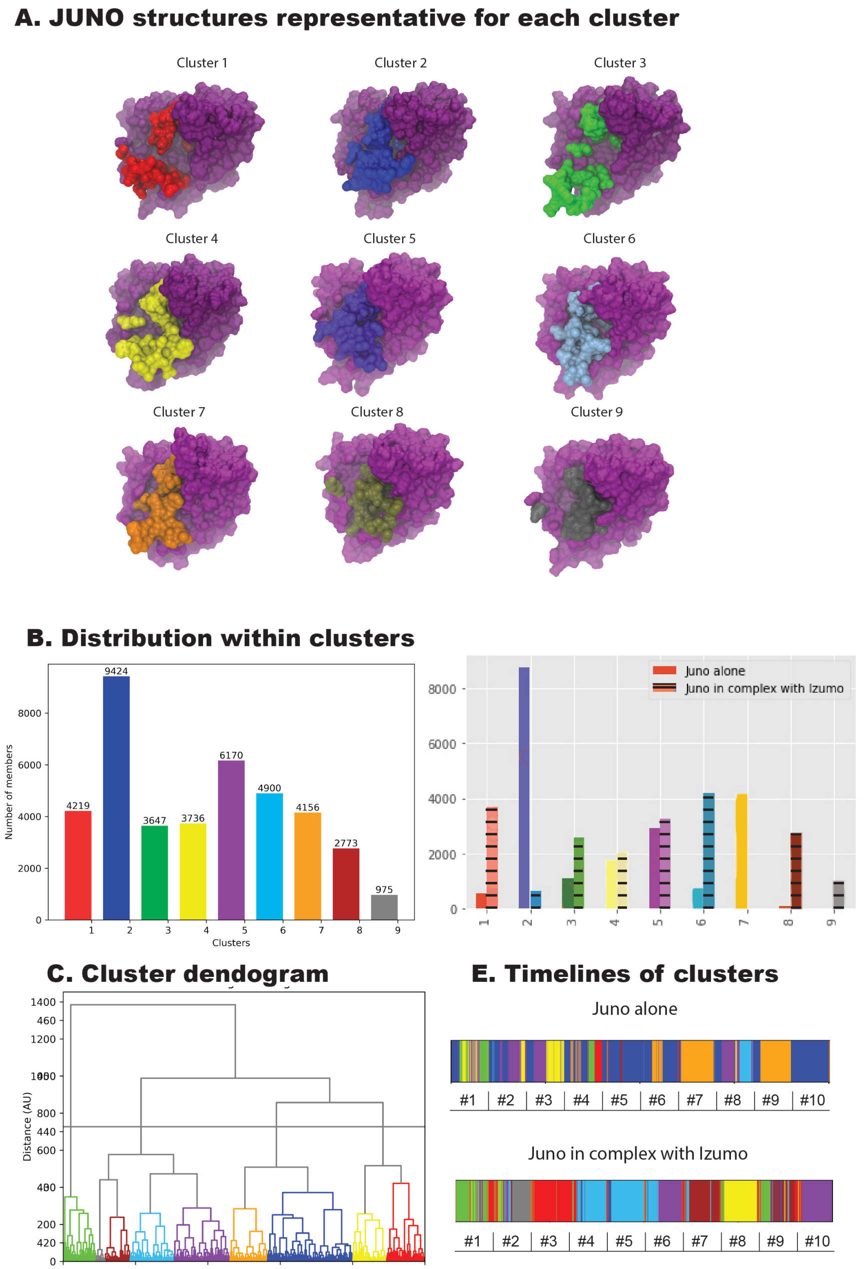

Fig. S18: ***Clustering of JUNO's structures.*** *A. The frames showing representative structure for each cluster differ the most around the putative folate binding pocket. Residues around the pocket with biggest RMSD between representative frames are colored. The clustering was done on trajectories of JUNO alone and JUNO from the complex with IZUMO1(20000 frames each set). B,C. Most of the clusters with intermediate access to the folate binding pocket show similar distribution between simulations alone and in the complex ; D.Timelines represent all 10 runs in consecutive order. Clustering done using TTClust. [12].*

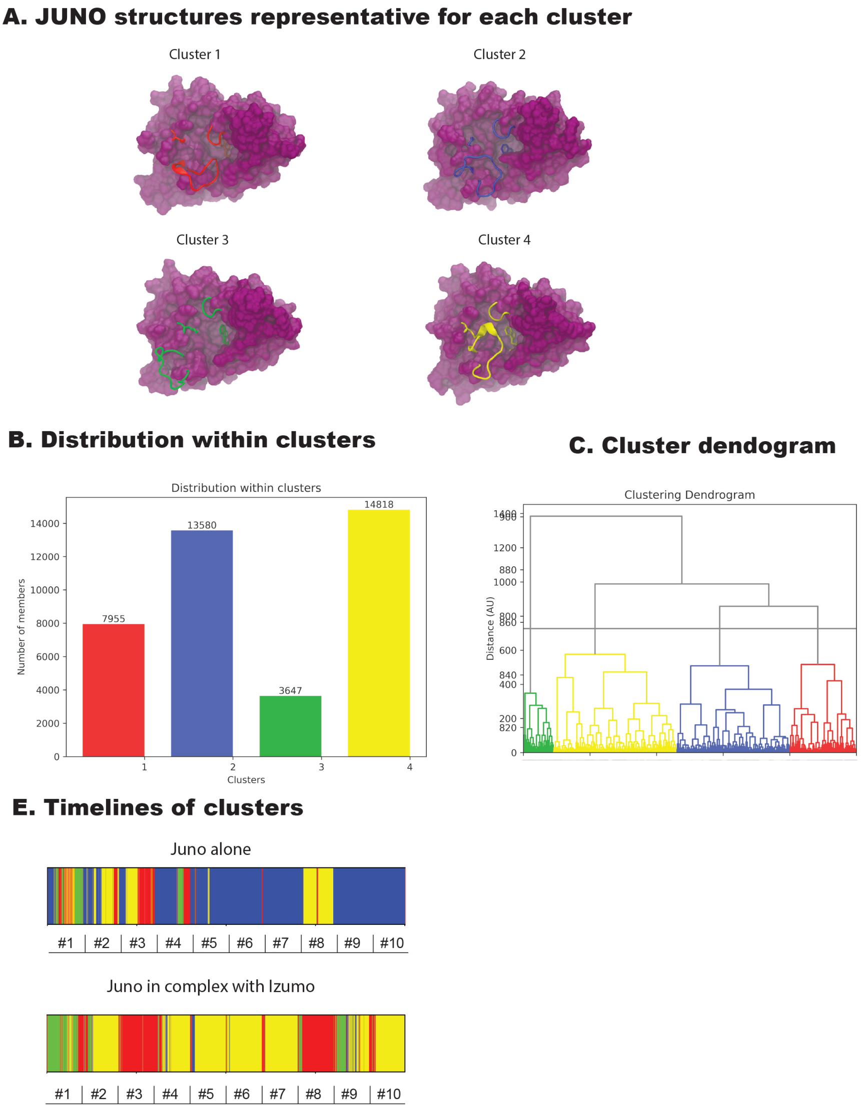

Fig. S19: ***Clustering of JUNO's structure into 4 groups.*** *A. Representative conformations of JUNO structure associated with each cluster. B,C. Distribution of frames withing clusters. Blue color corresponds to fully closed access to the pocket. Red and yellow show loop conformations, where the access to the inhibitory pocket is partially blocked. Green shows conformation of fully open state; C. Relative distance of clusters. D. Timelines represent all 10 runs in consecutive order of the JUNO and JUNO-IZUMO1 complex.*

Contact area analysis

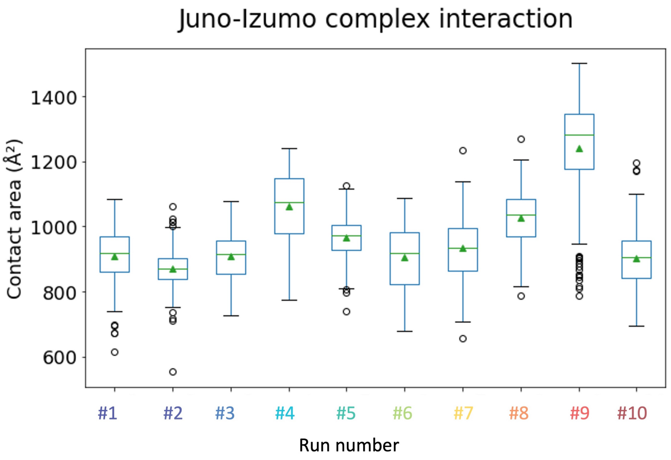

Fig. S20: ***Contact area between JUNO and IZUMO1 in equilibrium MD simulations*** *Each box plot represents one run. Green triangles mark the average, green lines mark the median.*

**run nb**	run 1	run 2	run 3	run 4	run 5	run 6	run 7	run 8	run 9	run 10
**mean**	910.5	870.8	909.3	1061.4	966.5	906.8	933.5	1027.9	1240.8	901.9
**std**	82.0	6.7	71.0	99.8	62.7	98.5	95.6	82.5	152.4	89.0
**min**	615.6	554.3	726.9	773.9	739.7	679.1	655.5	787.5	787.7	694.2
**max**	1085.3	1063.2	1079.2	1242.8	1127.6	1089.4	1234.1	1271.6	1501.8	1196.5

Table S2: ***Summary of the contact area values variations per run for JUNO-IZUMO1 complex (WT) simulations as sum.*** *See also Figure S20.*

Exemplary timelines of JUNO-IZUMO1 interactions

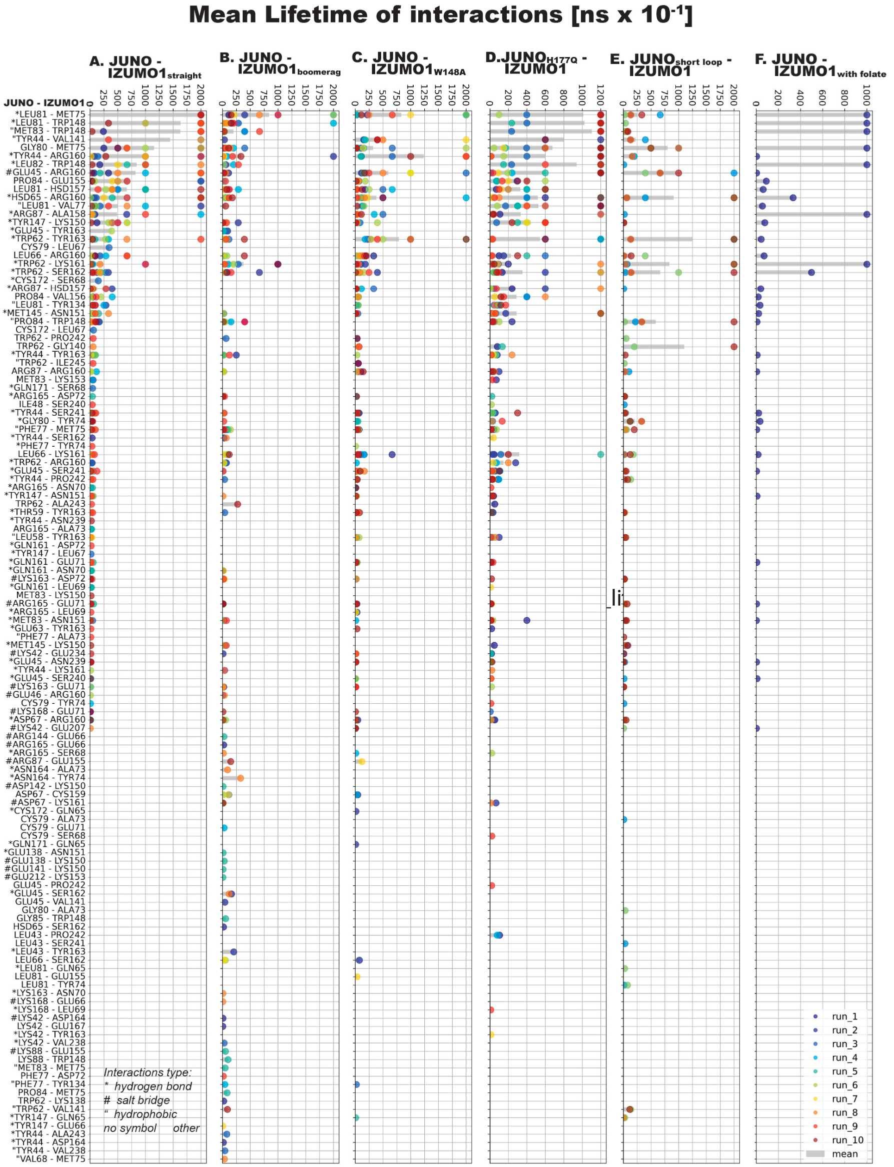

Fig. S21: ***Mean lifetimes of JUNO-IZUMO1 interactions as derived from MD simulations.*** *(Caption on the following page.)*

Fig. S21: ***Mean lifetimes of JUNO-IZUMO1 interactions as derived from MD simulations.*** *All interactions with PyContact score 1 in each run are shown and sorted according to the average life time across all runs for the WT. A. WT JUNO-IZUMO1 complex. 17 interactions have been formed, at least temporarily in all the runs: TYR44-ARG160, GLU45-ARG160, GLU45-SER241, TRP62-LYS161, TRP62-SER162, TRP62-TYR163, HSD65-ARG160, GLY80-MET75, LEU81-MET75, LEU81-TYR134, LEU81-TRP148, LEU81-HSD157, LEU82-TRP148, MET83-TRP148, PRO84-GLU155, TYR147-LYS150, ARG165-GLU71. B. JUNO-IZUMO1 in the "boomerang" conformation. Notably the average interaction time for previously stable interactions dropped and many residues previously at distance from the binding interface are now involved in inter-proteins interactions. C. Complex of WT JUNO with mutated IZUMO1 (W148A). D. Complex of mutated JUNO (H177Q) with WT IZUMO1. E. Complex of JUNO with short loop with WT IZUMO1 (5 runs only). F. JUNO-IZUMO1 complex upon spontaneous folate insertion (1 run).*

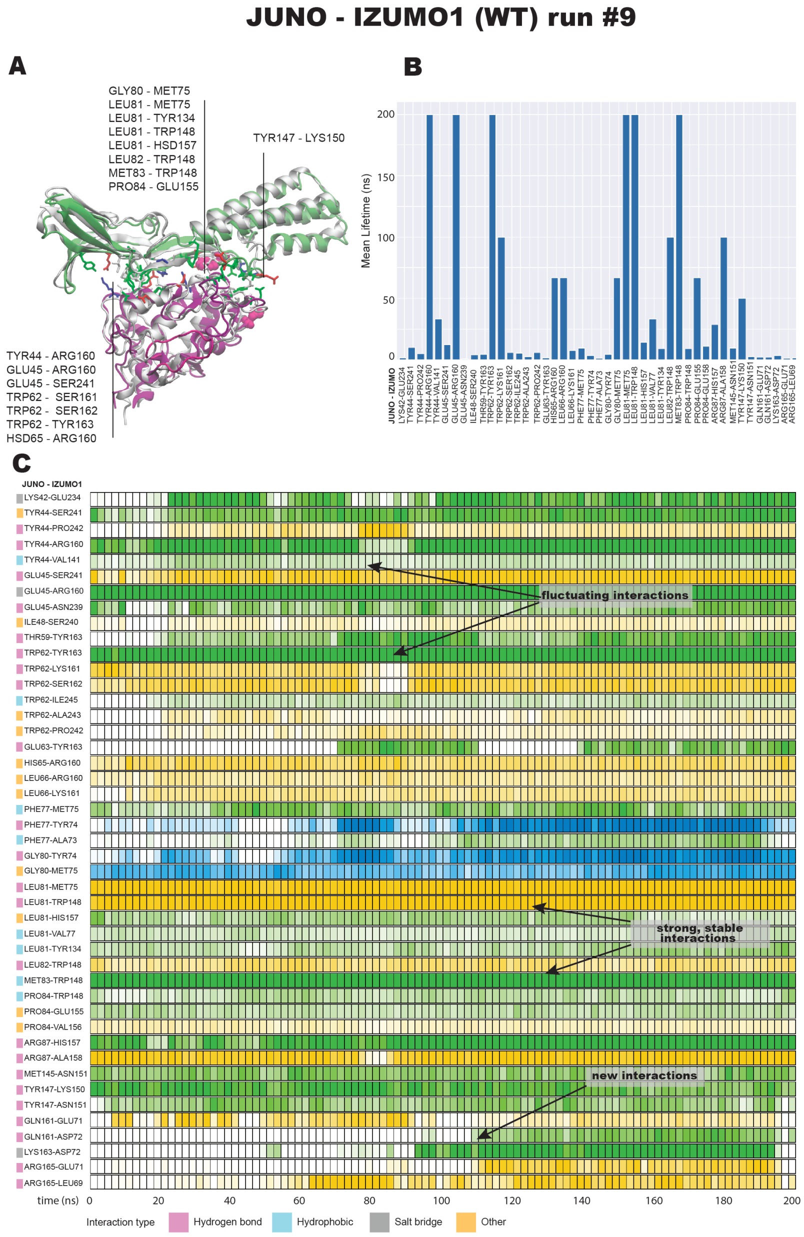

Fig. S22: ***Exemplary JUNO-IZUMO1 (WT) interactions as derived from MD simulations in run #9.*** *(Caption on the following page.)*

Fig. S22: ***Exemplary JUNO-IZUMO1 (WT) interactions as derived from MD simulations in run #9.*** *A. Structure of the JUNO (purple)-IZUMO1 (green) complex in the last frame (t = 200 ns) of the representative simulation with the highest number of interactions formed (45 interactions, run #9) compared to the crystal structure PDB 5JKE (in grey). Stick representation shows residues within 3.5 Å of the other protein. Regions of the protein modified in other runs are marked as pink spheres, on IZUMO1 it is TRP148, on JUNO it is HIS177 and inhibitory loop. Labeled residues (JUNO residue-IZUMO residue) correspond to the interactions formed at least temporarily in all runs. Water and ions are omitted for visualization purpose B. Mean lifetime of interactions (in frames) in run #9. C. Timeline of dynamic changes of the most prominent JUNO-IZUMO1 interactions in the run #9. Each box corresponds to the trajectory segment, their color signifies interaction type (green, side chain-side chain; yellow, side chain-backbone; blue, backbone-backbone) and intensity represents its strength. Interactions extracted using PyContact [6] with default settings: the threshold for hydrogen bond 2.5 Å and interaction mean score bigger than 1. Total simulation time was 200 ns (2000 frames).*

JUNO - IZUMO1 (WT) run #7

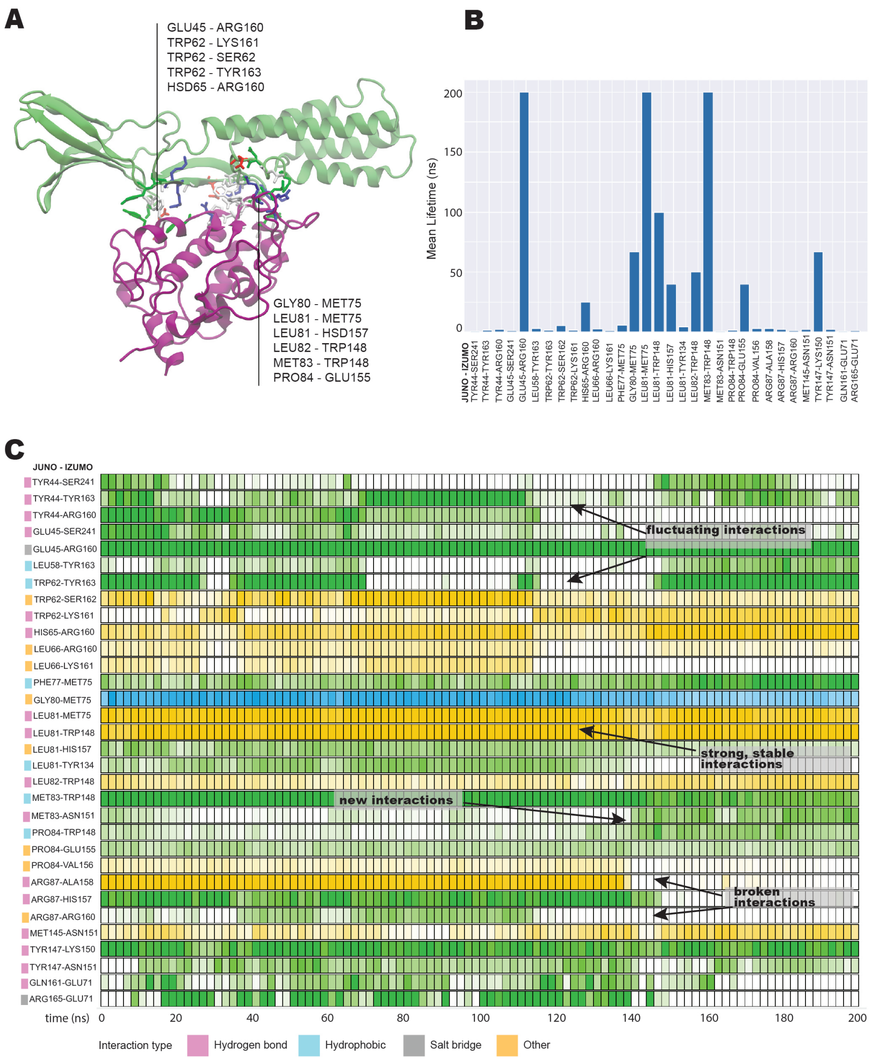

Fig. S23: ***JUNO-IZUMO1 (WT) interactions as derived from MD simulations in run #7.*** *(Caption on the following page.)*

Fig. S23: ***JUNO-IZUMO1 (WT) interactions as derived from MD simulations in run #7.*** *A. Structure of the JUNO (purple)-IZUMO1 (green) complex in the last frame (t = 200 ns) of the simulation that formed the smallest number of interactions (run #7). Stick representation shows residues within 3.0 Å of the other protein. Labeled residues (JUNO residue- IZUMO residue) correspond to the interactions formed at least temporarily in all runs of the WT complex (Figure S22A). Water and ions are omitted for visualization purpose. B. Mean lifetime of interactions (in frames) in run #7. C. Timeline of dynamic changes of the most prominent JUNO-IZUMO1 interactions in the run #7. Only 32 interactions were identified. Multiple bonds present in the starting conformation got broken in the simulation time. Each box corresponds to the trajectory segment, their color signifies interaction type (green, side chain-side chain; yellow, side chain-backbone; blue, backbone-backbone) and intensity represents its strength. Interactions extracted using PyContact [6] with default settings: threshold for hydrogen bond 2.5 Å and interaction mean score bigger than 1. Total simulation time was 200 ns (2000 frames).*

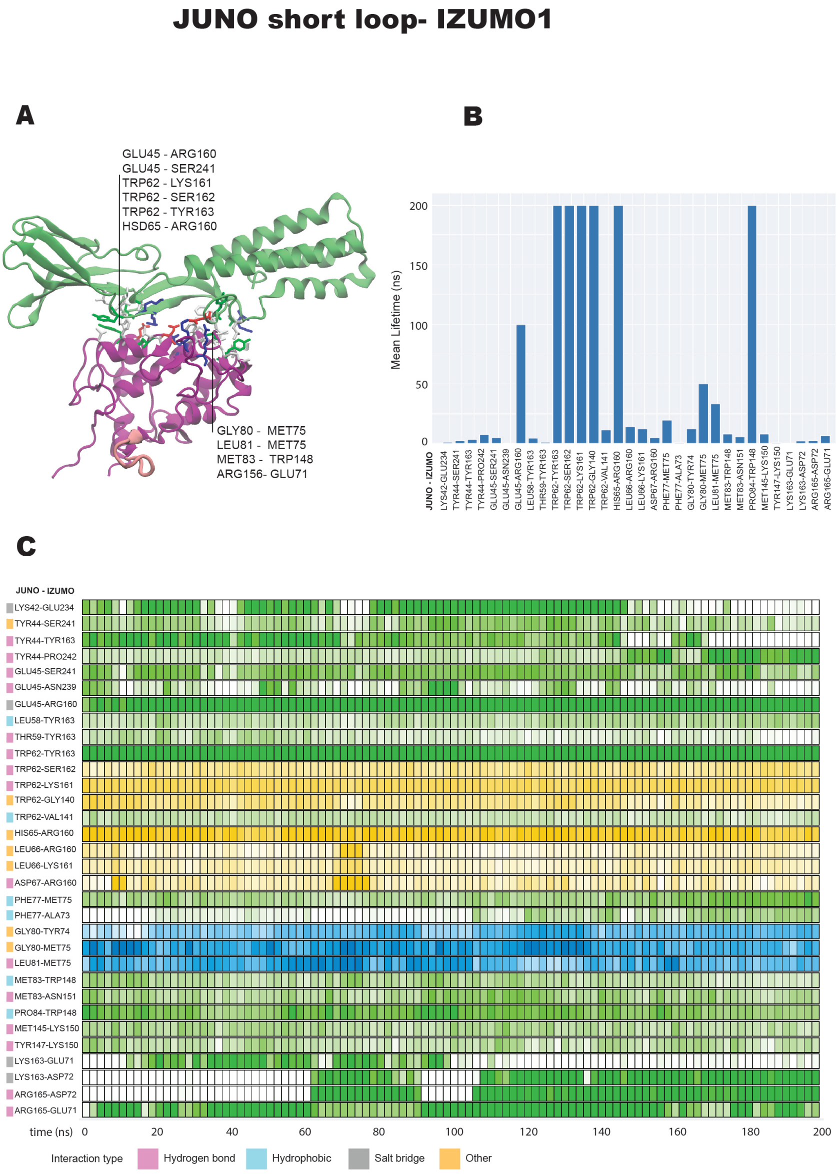

Fig. S24: ***JUNO$_{shortloop}$-IZUMO1 interactions as derived from MD simulations.*** *(Caption on the following page.)*

Fig. S24: ***JUNO$_{shortloop}$-IZUMO1 interactions as derived from MD simulations.*** *A. Final frame (t = 200 ns) of the representative simulation run #5. Small loss of contacts occurred in the binding interface compared to the wild type protein. Partial secondary structure loss can be noted on the N-terminal area of JUNO. Short loop is marked in pink. Stick representation shows residues within 3.0 Å of the other protein. Labeled residues (JUNO residue- IZUMO residue) correspond to the interactions formed at least temporarily in all runs of the WT complex (Figure S22, A). Water and ions are omitted for visualization purpose. B. Mean lifetime of interactions (in frames). C. Timeline of dynamic changes of the most prominent interactions. Each box corresponds to the trajectory segment, their color signifies interaction type (green, side chain-side chain; yellow, side chain-backbone; blue, backbone-backbone) and intensity represents its strength. Interactions extracted using PyContact [6] with default settings: threshold for hydrogen bond 2.5 Å and interaction mean score bigger than 1. Total simulation time was 200 ns (2000 frames).*

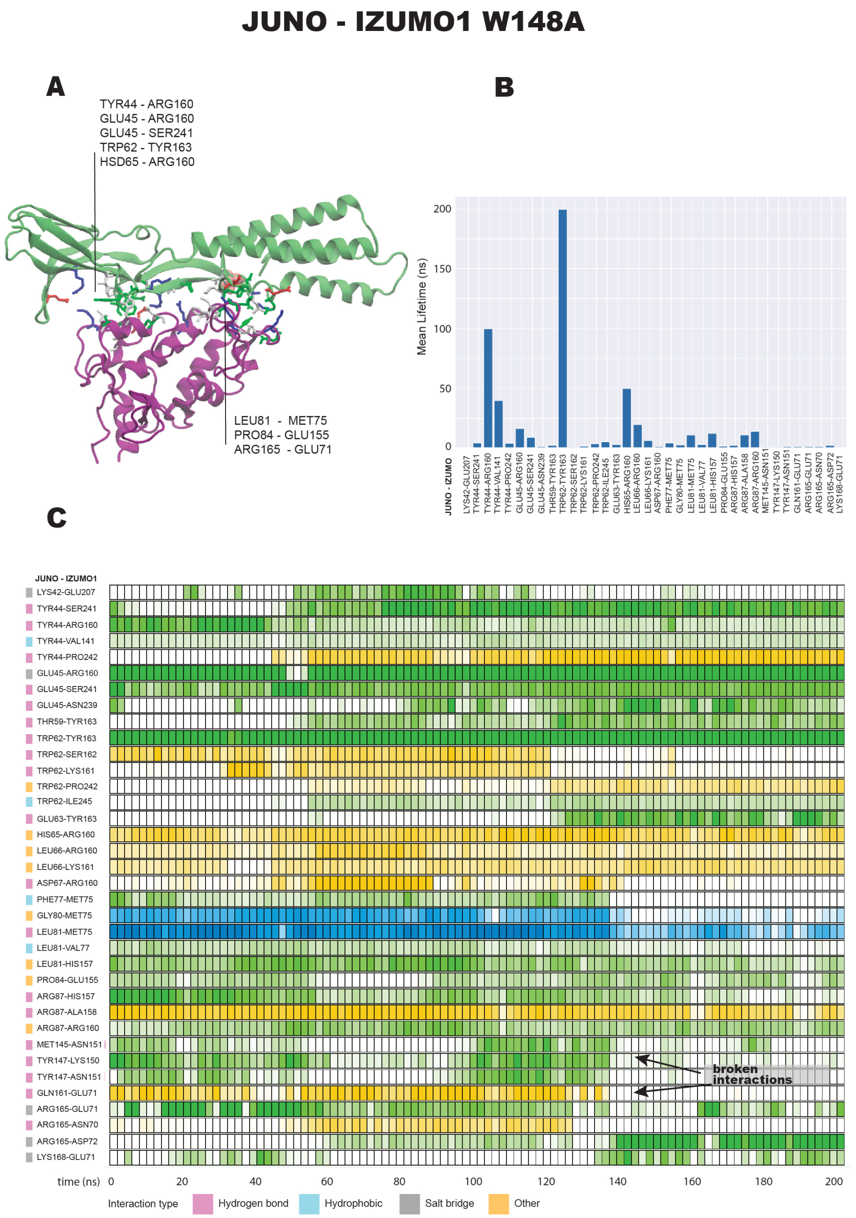

Fig. S25: ***JUNO-IZUMO1*** $_{W148A}$ ***interactions as derived from MD simulations.*** *(Caption on the following page.)*

Fig. S25: *JUNO-IZUMO1$_{W148A}$ interactions as derived from MD simulations.* *A. Final frame (t = 200 ns) of the representative simulation of the modified complex in run #10 (for the simulation trajectory, see Movie S4). Mutated residue 148 is colored pink. Stick representation shows residues within 3.0 Å of the other protein. Labeled residues (JUNO residue- IZUMO residue) correspond to the interactions formed at least temporarily in all runs of the WT complex (Figure S22, A). Water and ions are omitted for visualization purpose. Stick representation shows residues within 3.0 Å of the other protein. Residues labeled correspond to interactions in the snapshot, that are present at least temporarily in all runs of the wild type complex (Figure S22A). Water and ions are omitted for visualization purpose; B. Mean lifetime of interactions. C. Timeline of dynamic changes of the most prominent interactions. W148A mutation of IZUMO1 has been shown to abolish JUNO-IZUMO1 binding [9, 13]. In our 200 ns of simulation starting from the known bound structure of the complex with only this one point mutation, the gradual loss of the interactions can be observed. Each box corresponds to the trajectory segment, their color signifies interaction type (green, side chain-side chain; yellow, side chain-backbone; blue, backbone-backbone) and intensity represents its strength. Interactions extracted using PyContact [6] with default settings: threshold for hydrogen bond 2.5 Å and interaction mean score bigger than 1. Total simulation time was 200 ns (2000 frames).*

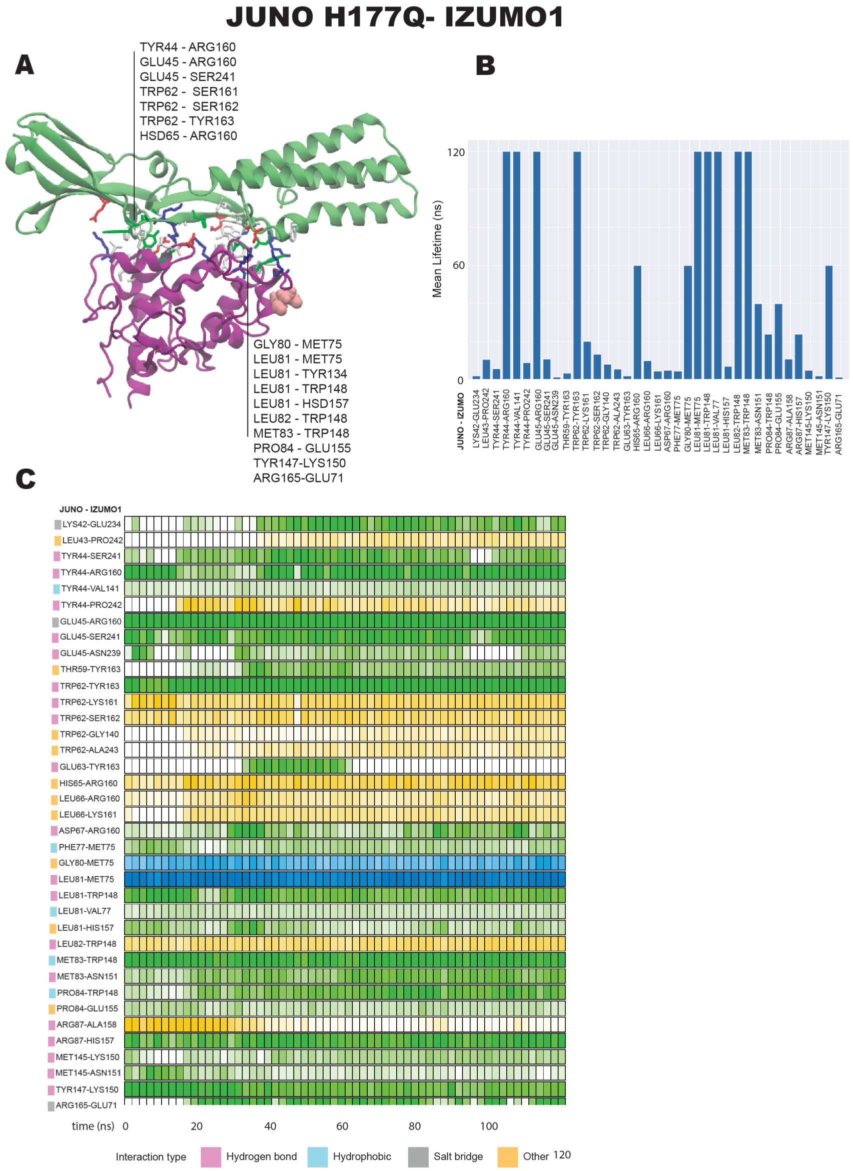

Fig. S26: ***JUNO$_{H177Q}$-IZUMO1 interactions as derived from MD simulations.*** *(Caption on the following page.)*

Fig. S26: *JUNO$_{H177Q}$-IZUMO1 interactions as derived from MD simulations.* *A. Final frame (t = 120 ns) of the representative simulation of the modified complex in run #1 (for the simulation trajectory, see Movie S5). Stick representation shows residues within 3.0 Å of the other protein. Labeled residues correspond to interactions (JUNO residue- IZUMO residue) in the snapshot, that are present at least temporarily in all runs of the wild type complex (Figure S22A). Water and ions are omitted for visualization purpose; B. Mean lifetime of interactions represented as number of frames. C. Timeline of dynamic changes of the most prominent interactions. H177Q mutation of JUNO has shown an impact on fertilization failure in in vitro patients [14]. This residue is not in direct contact in with IZUMO1 binding interface and in our equilibrium simulations we also did not observe any allosteric effect on the JUNO-IZUMO1 binding. Binding pattern was indistinguishable from the wild type proteins. Each box corresponds to the trajectory segment, their color signifies interaction type (green, side chain-side chain; yellow, side chain-backbone; blue, backbone-backbone) and intensity represents its strength. Interactions extracted using PyContact [6] with default settings: threshold for hydrogen bond 2.5 Å and interaction mean score bigger than 1. Total simulation time was 120 ns (1200 frames).*

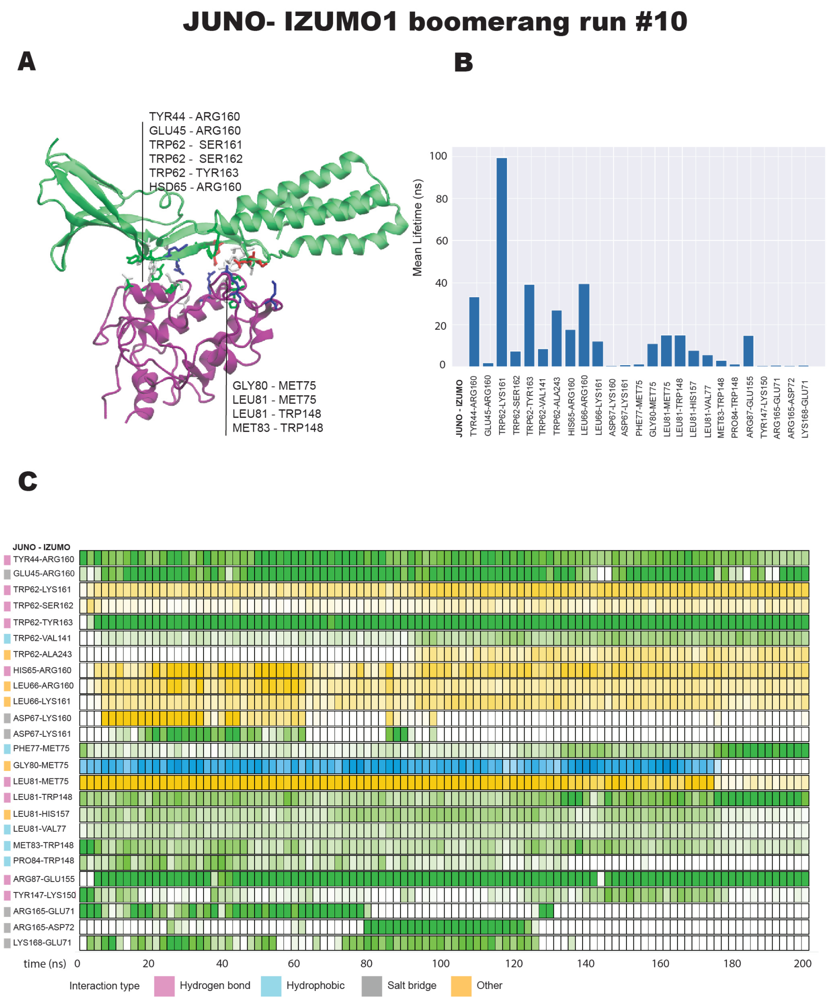

Fig. S27: ***JUNO-IZUMO1$_{boomerang}$* interactions as derived from MD simulations in run #10)** *A. Final frame of the simulation of the complex (t = 200 ns) in run #10. Stick representation shows residues within 3.0 Å of the other protein. Labeled residues (JUNO residue- IZUMO residue) correspond to the interactions formed at least temporarily in all runs of the WT complex (Figure S22, A). Water and ions are omitted for visualization purpose; B. Mean lifetime of interactions. C. Timeline of dynamic changes of the most prominent interactions. Each box on the plot corresponds to the trajectory segment, their color signifies interaction type (green, side chain-side chain; yellow, side chain-backbone; blue, backbone-backbone) and intensity represents its strength. Interactions extracted using PyContact [6] with default settings: threshold for hydrogen bond 2.5 Å and interaction mean score bigger than 1. Total simulation time was 200 ns (2000 frames)*

JUNO- IZUMO1 boomerang run #5

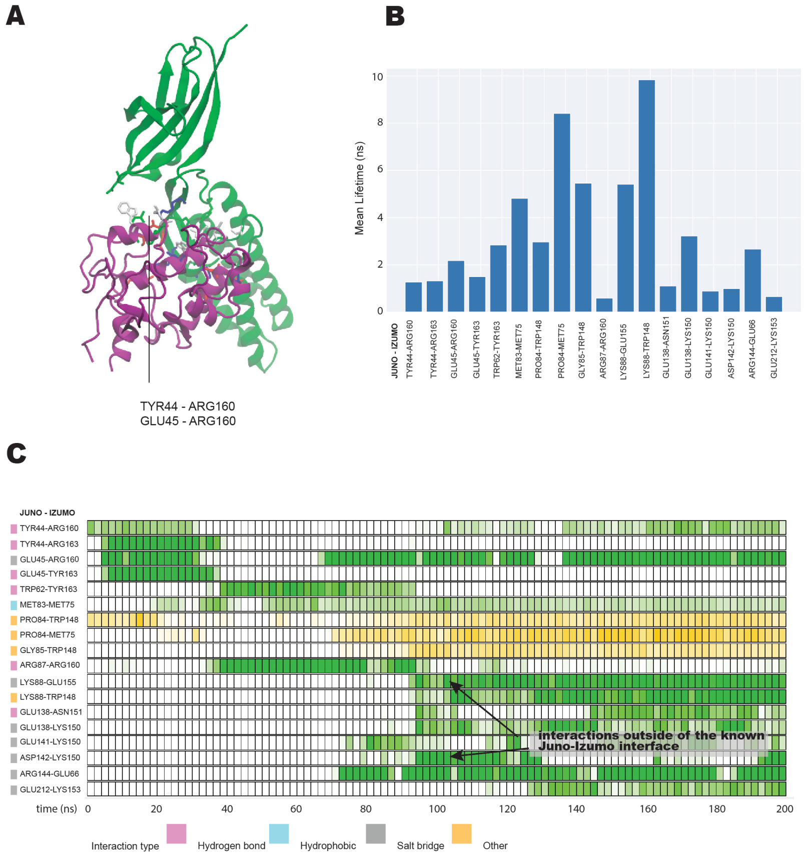

Fig. S28: ***JUNO-IZUMO1*$_{boomerang}$ *interactions as derived from MD simulations in run #5)*** *A. Final frame of the simulation of the complex (t = 200 ns) in run #5. Stick representation shows residues within 3.0 Å of the other protein. Labeled residues (JUNO residue- IZUMO residue) correspond to the interactions formed at least temporarily in all runs of the WT complex (Figure S22, A). Water and ions are omitted for visualization purpose; B. Mean lifetime of interactions. C. Timeline of dynamic changes of the most prominent interactions. Each box on the plot corresponds to the trajectory segment, their color signifies interaction type (green, side chain-side chain; yellow, side chain-backbone; blue, backbone-backbone) and intensity represents its strength. Interactions extracted using PyContact [6] with default settings: threshold for hydrogen bond 2.5 Å and interaction mean score bigger than 1. Total simulation time was 200 ns (2000 frames).*

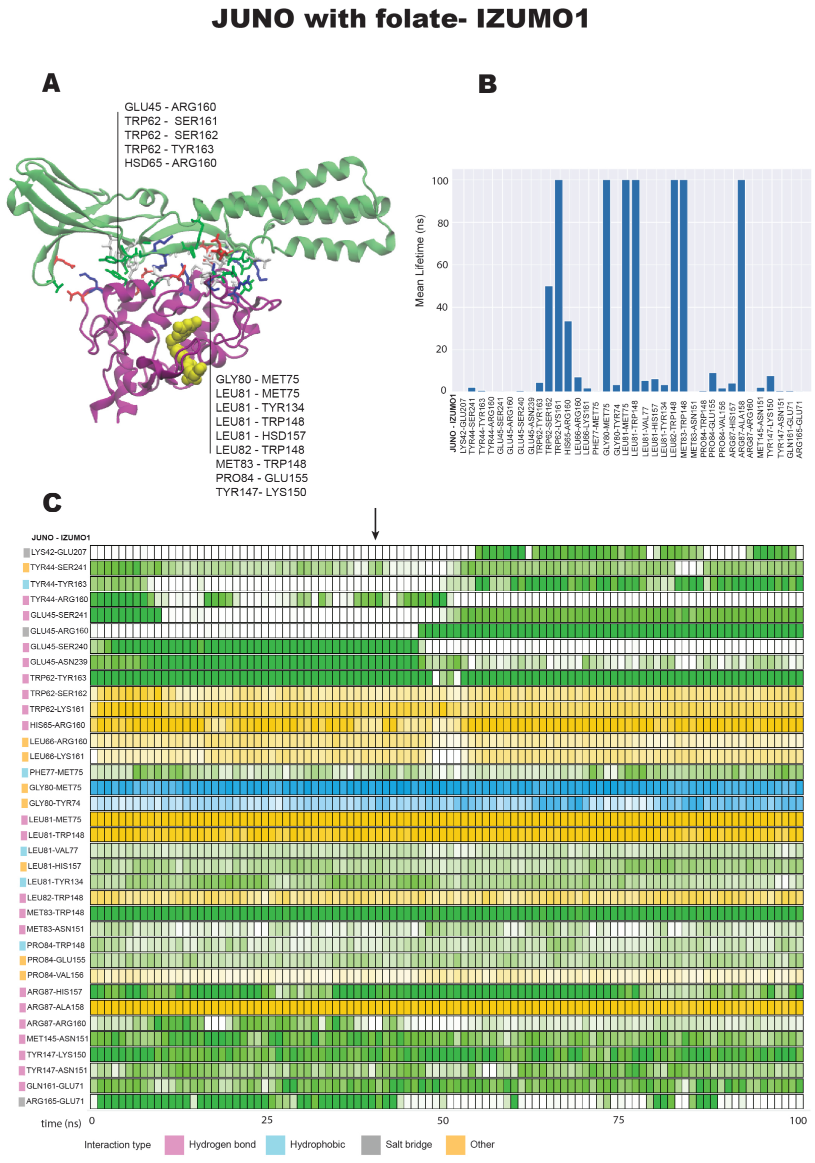

Fig. S29: ***JUNO-IZUMO1 interactions as derived from MD simulations of spontaneous folate binding.*** *(Caption on the following page.)*

Fig. S29: ***JUNO-IZUMO1 interactions as derived from MD simulations of spontaneous folate binding.*** *A. Final frame (t = 100 ns) of the simulation of the complex upon folate binding. Folate is marked in yellow. Stick representation shows residues within 3.0 Å of the other protein. Labeled residues (JUNO residue- IZUMO residue) correspond to the interactions formed at least temporarily in all runs of the WT complex (Figure S22, A). Water and ions are omitted for visualization purpose. B. Mean lifetime of interactions. C. Timeline of dynamic changes of the most prominent interactions. Each box on the plot corresponds to the trajectory segment, their color signifies interaction type (green, side chain-side chain; yellow, side chain-backbone; blue, backbone-backbone) and intensity represents its strength. Interactions extracted using PyContact [6] with default settings, threshold for hydrogen bond 2.5 Å and interaction mean score bigger than 1. Total simulation time was 100 ns (5000 frames).*

Heavy ion binding to JUNO and IZUMO1

Heavy metal binding sites were determined using the online Metal Ion-Binding (MIB) Site Prediction and Docking Server [15, 16] on structures of IZUMO1 "boomerang" (Figure S30) and JUNO from the complex (Figure S31). Resulting predictions of top scoring positions for Hg^{2+}, Cd^{2+}, Zn^{2+} and Cu^{2+} ions are shown.

Heavy ion	Effective ionic radii [pm]
**Hg^{2+}**	119
**Pb^{2+}**	119
**Cd^{2+}**	95
**Zn^{2+}**	74
**Cu^{2+}**	73
Cu^{+}	91
Pb^{4+}	77.5
Fe^{2+}	92
Fe^{3+}	78.5
Cu^{2+}	73

Table S3: ***Heavy metals known to impact male infertility are given in bold [17], and all are given with their effective ionic radii.***

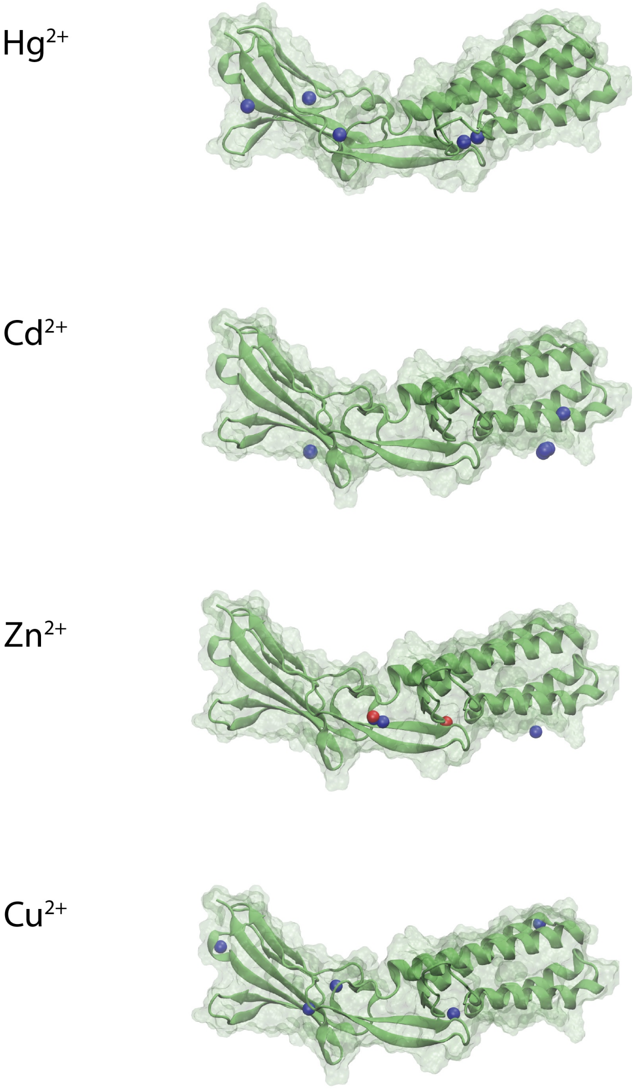

Fig. S30: ***Predicted heavy ion binding sites for IZUMO1 in the "boomerang" conformation.*** *Predictions made with MIB [16]. Red color marks which binding sites were used in the simulations.*

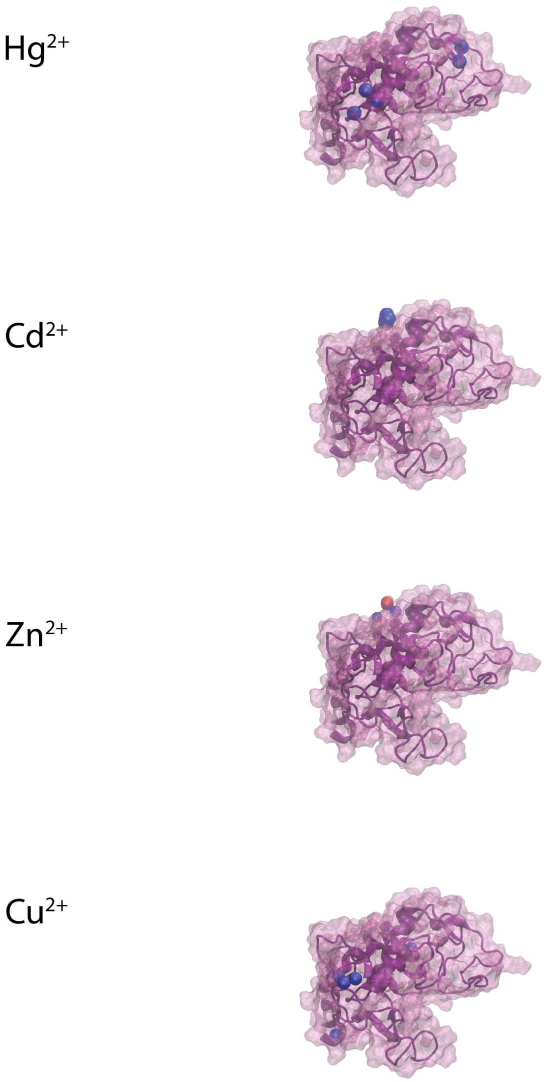

Fig. S31: ***Predicted heavy ion binding sites for JUNO.*** *Predictions were made with MIB [16].*

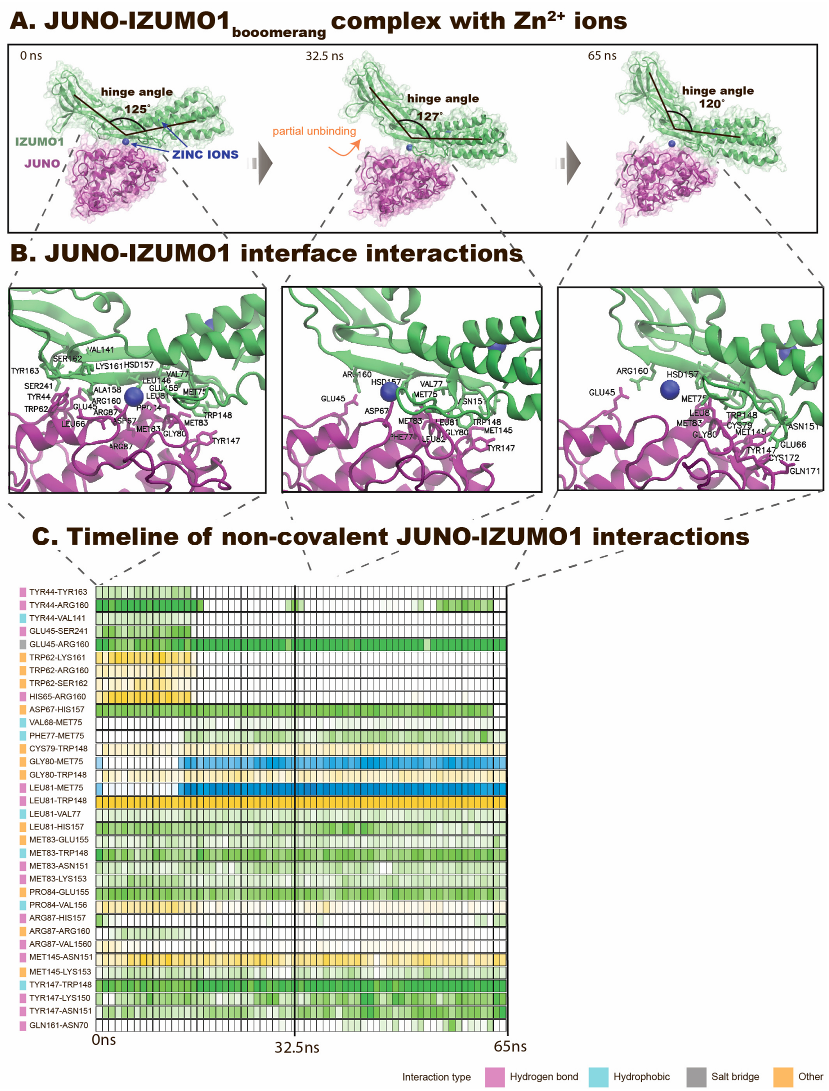

Fig. S32: *Snapshots how the JUNO-IZUMO1 complex equilibrates after* Zn^{2+} *ions were inserted into the predicted zinc binding sites of* $IZUMO1_{boomerang}$. *(Caption on the following page.)*

Fig. S32: ***Snapshots how the JUNO-IZUMO1 complex equilibrated after Zn^{2+} ions were inserted into the MIB-predicted zinc binding sites of IZUMO1$_{boomerang}$.*** *A. During this preliminary 62 ns MD simulation, a progressive reduction of the contact area between JUNO and IZUMO1 occurred, while the Zn^{2+} ions remained in their positions. Measured hinge angle shows that IZUMO1 remains in the "boomerang" conformation. B. Gradual loss of interactions at the interface suggests that this short trajectory captures the early phase of JUNO-IZUMO1 dissociation. To conduct this simulation the complex was prepared by superposing previously predicted structure of IZUMO1$_{boomerang}$-Zn^{2+} ions system (see also Figure S30) with the crystal structure of the complex. JUNO position was than adjusted to account for the sterical clashes. Simulation were performed in explicit water with the same equilibration protocol used for other systems and simulated for 62 ns. Water was omitted for visualization purposes. C.Timeline of dynamic changes of the most prominent JUNO-IZUMO1 interactions in this preliminary simulation. Each box corresponds to the trajectory segment, their color signifies interaction type (green, side chain-side chain; yellow, side chain-backbone; blue, backbone-backbone) and intensity represents its strength. Interactions extracted using PyContact [6] with default settings: threshold for hydrogen bond 2.5 Å and interaction mean score bigger than 0.2. Total simulation time was 65 ns.)*

Glycosylated JUNO-IZUMO1 complex

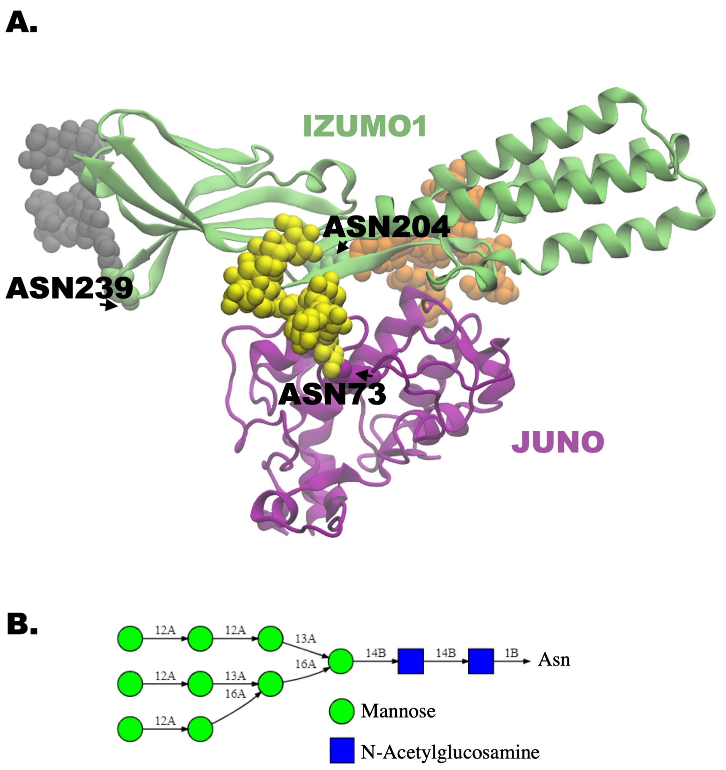

Fig. S33: *Modeled structure of the glycosylated JUNO-IZUMO1 complex*. *A. Location of the N-glycosylation sites on JUNO (ASN73) and IZUMO1 (ASN204, ASN239) [9, 13]. Grey, yellow and orange marks the respective glycans. B. Type of the glycans used for modeling. CHARMM GUI was used to setup the system [18, 19].*

Movie Legends

Movie S1: ***Exemplary MD trajectory of the JUNO (purple)-IZUMO1 (green) complex simulation (run #9).*** *The contact surface between two proteins increases in the course of the simulation. We can see new interactions formed between residues, that are outside of the binding interface identified by the crystal structure. Essential spots, modified in other simulations are marked as follows: inhibitory loop is pink, mutated residues on IZUMO1 (W148) and on JUNO (H177) are represented as spheres. Residues within 3.5 Å of the other protein are represented as sticks and colored according to their type (green: polar, red: acidic, blue: basic, white: non-polar). Simulation time was 200 ns. Water and ions were removed for visualization purpose. Movie rendered in VMD [20, 21].*

Movie S2: ***Exemplary MD trajectory of the JUNO (purple)-IZUMO1 (green) complex simulation (run #7).*** *In this run, the contact area between the two proteins is overall smaller than in other other runs, going along with the higher flexibility of the binding interface and partial straightening of the IZUMO1. Essential spots, modified in other simulations are marked as follows: inhibitory loop is pink, mutated residues on IZUMO1 TRP148 and on JUNO HIS177 are represented as spheres. Residues within 3 Å of the other protein are represented as sticks and colored according to their type (green:polar, red: acidic, blue: basic, white: non-polar). Simulation time was 200 ns. Water and ions were removed for visualization purposes. Movie rendered in VMD [20, 21].*

Movie S3: ***Exemplary MD trajectory of the $JUNO_{shortloop}$ (purple)-IZUMO1 (green) complex simulation.*** *The inhibitory loop has been shortened by deletion of the residues 119-124. The differences in the dynamics of the binding interface when compared to the WT complex are subtle and hard to spot without the quantitative assessment (see also Figure S6). The short loop is colored pink. Residues within 3 Å of the other protein are represented as sticks and colored according to their type (green:polar, red: acidic, blue: basic, white: non-polar). Simulation time was 200 ns. Water and ions were removed for visualization purposes. Movie rendered in VMD [20, 21].*

Movie S4: ***Exemplary MD trajectory of the mutated JUNO (purple)-IZUMO1$_{W148A}$ (green) complex.*** *Mutated residue is shown as pink spheres. The that lack of the tryptophan disrupted the interaction network and caused step-wise loss of contacts in the binding interface. Residues within 3 Å of the other protein are represented as sticks and colored according to their type (green:polar, red: acidic, blue: basic, white: non-polar). Simulation time was 200 ns. Water and ions were removed for visualization purposes. Movie rendered in VMD [20, 21].*

Movie S5: ***Exemplary MD trajectory of the mutated JUNO$_{H177Q}$ (purple)-IZUMO1 (green) complex simulation.*** *The binding interface is unaffected by this change. Mutated residue is shown as pink spheres. Residues within 3 Å of the other protein are represented as sticks and colored according to their type (green: polar, red: acidic, blue: basic, white: non-polar). Simulation time was 120 ns. Water and ions were removed for visualization purposes. Movie rendered in VMD [20, 21].*

Movie S6: ***Exemplary MD trajectory of the JUNO (purple)-IZUMO1$_{boomerang}$ (light green) complex simulation (run #10) .*** *Repeated simulation runs yield different outcomes: in the one shown on this video, the initial partial contact is enough to recover missing interactions. Residues within 3 Å of the other protein are represented as sticks and colored according to their type (green: polar, red: acidic, blue: basic, white: non-polar). Simulation time was 200 ns. Water and ions were removed for visualization purposes. Movie rendered in VMD [20, 21].*

Movie S7: ***Exemplary MD trajectory of JUNO (purple)-IZUMO1$_{boomerang}$ (light green) complex simulation (run #5).*** *Repeated simulation runs yielded different outcomes: in the one shown in here, the conformation of IZUMO1 and its relative position to JUNO changed completely. Residues within 3 Å of the other protein are represented as sticks and colored according to their type (green: polar, red: acidic, blue: basic, white: non-polar). Simulation time was 200 ns. Water and ions were removed for visualization purposes. Movie rendered in VMD [20, 21].*

Movie S8: ***Exemplary MD trajectory of the JUNO (purple)-IZUMO1 (green) complex interactions upon spontaneous folate (yellow) binding.*** *Binding interface seems largely unaffected by the folate insertion, however, small adjustments happen within the first few ns and new JUNO-IZUMO1 interactions are formed (see also Figure S29). Residues within 3 Å of the other protein are represented as sticks and colored according to their type (green:polar, red: acidic, blue: basic, white: non-polar). Simulation time is 100 ns. Water and ions were removed for visualization purposes. Movie rendered in VMD [20, 21].*

Movie S9: ***Exemplary MD trajectories of the IZUMO1 bending motion in different simulation conditions.*** *For easier comparison, the initial and final frames are always displayed. Independent of the starting conformation, IZUMO1 freely shifts between different hinge angles, either when alone or when in complex with JUNO. Once, zinc ions are inserted (blue), IZUMO1 was preferentially found in the "boomerang" conformation. Water and ions were removed for visualization purposes. Movie rendered in VMD [20, 21].*

Movie S10: ***Exemplary MD trajectories of JUNO fluctuations in different simulation conditions.*** *Notice, how the inhibitory loop marked in red bent and turned around the entrance of the binding pocket. When JUNO was in complex with IZUMO1 the loop stretched into the solvent and did not block the entrance to the binding pocket. Simulation time was 200 ns. The protein was superposed to the first frame to remove diffusion. Water and solvent ions were removed for visualization purpose. Movies rendered in VMD [20, 21].*

Movie S11: ***Exemplary MD trajectory of the "refined" folate binding to JUNO in complex with IZUMO1 starting from a partially bound pose.*** *Folate placed at the entrance to the binding pocket briefly screened the surface of JUNO and than slide into the cavity. Once bound remains inside and achieved the pose very similar to the known crystal structure of folate inserted into the folate receptor alpha (see also Figure S2). Simulation duration was 20 ns. Water and ions were removed for visualization purposes. Movie rendered in VMD [20, 21].*

Movie S12: ***Exemplary MD trajectory of the spontaneous folate binding to JUNO in complex with IZUMO1 in unbiased simulations.*** *Folate was allowed to diffuse freely in solution. In this successful simulation it attached to the surface of JUNO within few nanoseconds and guided, inserted ito the binding pocket (see also Figure 5 in the main text). Once inside, it explored the large binding pocket and adjusted its conformation. Consequently, the inhibitory loop partially blocked the exit. Simulation time was 100 ns. Water and ions were removed for visualization purposes. Movie rendered in VMD [20, 21].*

Movie S13: ***Exemplary MD trajectory of the JUNO alone with folate when starting the simulation from a partially bound pose.*** *Folate unbound from the surface of JUNO to subsequently screen its surface. The inhibitory loop rather tightly bound to JUNO's surface made it impossible for folate to slide inside the binding pocket. Water and ions were removed for visualization purposes. Movie rendered in VMD [20, 21].*

References

[1] Bianchi, E., Doe, B., Goulding, D., Wright, G.J.: Juno is the egg Izumo receptor and is essential for mammalian fertilization. Nature **508**(7497), 483–487 (2014). https://doi.org/10.1038/nature13203

[2] Han, L., Nishimura, K., Sadat Al Hosseini, H., Bianchi, E., Wright, G.J., Jovine, L.: Divergent evolution of vitamin b9 binding underlies juno-mediated adhesion of mammalian gametes. Current Biology **26**(3), 100–101 (2016). https://doi.org/10.1016/j.cub.2015.12.034

[3] Chen, C., Ke, J., Edward Zhou, X., Yi, W., Brunzelle, J.S., Li, J., Yong, E.L., Xu, H.E., Melcher, K.: Structural basis for molecular recognition of folic acid by folate receptors. Nature **500**(7463), 486–489 (2013). https://doi.org/10.1038/nature12327

[4] Petrova, J., Gocheva, G., Ivanova, N., Iliev, S., Atanasova, B., Madjarova, G., Ivanova, A.: Molecular simulation of the structure of folate and antifolates at physiological conditions. Journal of Molecular Graphics and Modelling **87**, 172–184 (2019). https://doi.org/10.1016/j.jmgm.2018.11.018

[5] Schaber, E.N., Ivanova, N., Iliev, S., Petrova, J., Gocheva, G., Madjarova, G., Ivanova, A.: Initial Stages of Spontaneous Binding of Folate-Based Vectors to Folate Receptor - α Observed by Unbiased Molecular Dynamics (2021). https://doi.org/10.1021/acs.jpcb.1c00488

[6] Scheurer, M., Rodenkirch, P., Siggel, M., Bernardi, R.C., Schulten, K., Tajkhorshid, E., Rudack, T.: PyContact: Rapid, Customizable, and Visual Analysis of Noncovalent Interactions in MD Simulations. Biophysical Journal **114**(3), 577–583 (2018). https://doi.org/10.1016/j.bpj.2017.12.003

[7] Gocheva, G., Ivanova, N., Iliev, S., Petrova, J., Madjarova, G., Ivanova, A.: Characteristics of a Folate Receptor-α Anchored into a Multilipid Bilayer Obtained from Atomistic Molecular Dynamics Simulations. Journal of Chemical Theory and Computation **16**(1), 749–764 (2020). https://doi.org/10.1021/acs.jctc.9b00872

[8] Wibowo, A.S., Singh, M., Reeder, K.M., Carter, J.J., Kovach, A.R., Meng, W., Ratnam, M., Zhang, F., Dann, C.E.: Structures of human folate receptors reveal biological trafficking states and diversity in folate and antifolate recognition. Proceedings of the National Academy of Sciences **110**(38), 15180–15188 (2013) https://arxiv.org/abs/arXiv:1408.1149. https://doi.org/10.1073/pnas.1308827110

[9] Aydin, H., Sultana, A., Li, S., Thavalingam, A., Lee, J.E.: Molecular architecture of the human sperm IZUMO1 and egg JUNO fertilization complex. Nature **534**(7608), 562–565 (2016). https://doi.org/10.1038/nature18595

[10] Stuhrmann, H.B.: Small-angle scattering of X-rays. Progress In Crystal Growth And Characterization **18**(C), 1–19 (1989). https://doi.org/10.1016/0146-3535(89)90023-3

[11] Hub, J.S.: Interpreting solution X-ray scattering data using molecular simulations. Current Opinion in Structural Biology **49**, 18–26 (2018). https://doi.org/10.1016/j.sbi.2017.11.002

[12] Tubiana, T., Carvaillo, J.C., Boulard, Y., Bressanelli, S.: TTClust: A Versatile Molecular Simulation Trajectory Clustering Program with Graphical Summaries. Journal of Chemical Information and Modeling **58**(11), 2178–2182 (2018). https://doi.org/10.1021/acs.jcim.8b00512

[13] Ohto, U., Ishida, H., Krayukhina, E., Uchiyama, S., Inoue, N., Shimizu, T.: Structure of IZUMO1-JUNO reveals sperm-oocyte recognition during mammalian fertilization. Nature **534**(7608), 566–569 (2016). https://doi.org/10.1038/nature18596

[14] Yu, M., Zhao, H., Chen, T., Tian, Y., Li, M., Wu, K., Bian, Y., Su, S., Cao, Y., Ning, Y., Liu, H., Chen, Z.J.: Mutational analysis of IZUMO1R in women with fertilization failure and polyspermy after in vitro fertilization. Journal of Assisted Reproduction and Genetics **35**(3), 539–544 (2018). https://doi.org/10.1007/s10815-017-1101-5

[15] Lu, C.H., Lin, Y.F., Lin, J.J., Yu, C.S.: Prediction of metal ion-binding sites in proteins using the fragment transformation method. PLoS ONE **7**(6), 1–12 (2012). https://doi.org/10.1371/journal.pone.0039252

[16] Lin, Y.F., Cheng, C.W., Shih, C.S., Hwang, J.K., Yu, C.S., Lu, C.H.: MIB: Metal Ion-Binding Site Prediction and Docking Server. Journal of Chemical Information and Modeling **56**(12), 2287–2291 (2016). https://doi.org/10.1021/acs.jcim.6b00407

[17] Bhardwaj, J.K., Paliwal, A., Saraf, P.: Effects of heavy metals on reproduction owing to infertility. Journal of Biochemical and Molecular Toxicology **35**(8), 1–21 (2021). https://doi.org/10.1002/jbt.22823

[18] Jo, S., Kim, T., Iyer, V.G., Im, W.: CHARMM-GUI: A web-based graphical user interface for CHARMM. Journal of Computational Chemistry **29**(11), 1859–1865 (2008). https://doi.org/10.1002/jcc.20945

[19] Lee, J., Cheng, X., Swails, J.M., Yeom, M.S., Eastman, P.K., Lemkul, J.A., Wei, S., Buckner, J., Jeong, J.C., Qi, Y., Jo, S., Pande, V.S., Case,

D.A., Brooks, C.L., MacKerell, A.D., Klauda, J.B., Im, W.: CHARMM-GUI Input Generator for NAMD, GROMACS, AMBER, OpenMM, and CHARMM/OpenMM Simulations Using the CHARMM36 Additive Force Field. Journal of Chemical Theory and Computation **12**(1), 405–413 (2016). https://doi.org/10.1021/acs.jctc.5b00935

[20] Humphrey, W., Dalke, A., Schulten, K.: VMD – Visual Molecular Dynamics. Journal of Molecular Graphics **14**, 33–38 (1996)

[21] Stone, J.: An Efficient Library for Parallel Ray Tracing and Animation. Master's thesis, Computer Science Department, University of Missouri-Rolla (1998)
